# Functional and Structural Brain Changes Associated with Methamphetamine Abuse

**DOI:** 10.3390/brainsci2040434

**Published:** 2012-10-01

**Authors:** Reem K. Jan, Rob R. Kydd, Bruce R. Russell

**Affiliations:** 1 School of Pharmacy, Faculty of Medical and Health Sciences, University of Auckland, Private Bag 92019, Auckland 1142, New Zealand; Email: b.russell@auckland.ac.nz; 2 Centre for Brain Research, Faculty of Medical and Health Sciences, University of Auckland, Private Bag 92019, Auckland 1142, New Zealand; Email: r.kydd@auckland.ac.nz; 3 Department of Psychological Medicine, Faculty of Medical and Health Sciences, University of Auckland, Private Bag 92019, Auckland 1142, New Zealand

**Keywords:** addiction, cognitive control, decision-making, dopamine, grey matter, functional magnetic resonance imaging, methamphetamine, neurotoxicity, positron emission tomography, serotonin

## Abstract

Methamphetamine (MA) is a potent psychostimulant drug whose abuse has become a global epidemic in recent years. Firstly, this review article briefly discusses the epidemiology and clinical pharmacology of methamphetamine dependence. Secondly, the article reviews relevant animal literature modeling methamphetamine dependence and discusses possible mechanisms of methamphetamine-induced neurotoxicity. Thirdly, it provides a critical review of functional and structural neuroimaging studies in human MA abusers; including positron emission tomography (PET) and functional and structural magnetic resonance imaging (MRI). The effect of abstinence from methamphetamine, both short- and long-term within the context of these studies is also reviewed.

## 1. Epidemiology and Clinical Pharmacology of Methamphetamine Abuse

Abuse of the highly addictive synthetic psychostimulants, methamphetamine (MA) and its metabolite amphetamine, has become a global epidemic. During 2007, the estimated number of amphetamine and MA abusers reached 25 million globally, exceeding the total number of heroin and cocaine abusers [1]. The United Nations Office on Drugs and Crime estimated the number of people who abused MA to be 15–16 million during 2007, worldwide [1]. In 2008, between 13.7 and 52.9 million people aged 15–64 years were estimated globally to have used an amphetamine-type drug at least once in the past year and the number of MA seizures reached 19.3 metric tons worldwide [2]. 

The physiological and psychological effects of MA significantly outlast those of other psychostimulant drugs of abuse such as cocaine owing to MA’s considerably longer elimination half-life (8–13 h *vs.* 1–3 h for cocaine) and its high lipid solubility which allows for its rapid access to brain cells through the blood-brain barrier [3,4]. 

Apart from its effects on the cardiovascular system, intake of MA causes acute psychological and behavioural effects including positive feelings of euphoria, arousal and reduced fatigue and negative feelings of anxiety and paranoia [5]. The acute administration of MA can cause improvements in cognition including improved sustained attention, concentration and motor coordination. However, chronic MA abuse is associated with cognitive deficits in attention, executive function and working memory [6,7,8]. Overdose of MA can cause unpleasant psychological effects such as agitation, hallucination and psychosis [5]. Acute withdrawal from MA is often referred to as the “crash” as it is associated with depression-like symptoms including low mood, anxiety, irritability, fatigue and disturbed sleep as well as increased craving and cognitive impairment [9]. For a comprehensive review of the clinical pharmacological effects of MA, see Cruickshank and Dyer 2009 [5]. 

The mechanism of action of MA involves multiple pathways resulting in increased release and extracellular concentration of the monoamines dopamine (DA), noradrenaline (NA) and serotonin (5-HT) [10,11,12]. Similarly to cocaine, MA and other amphetamines increase extracellular DA levels by preventing the reuptake of DA into presynaptic terminals through blocking the DA transporter (DAT) [13,14] or decreasing its expression on the cell surface [15]. By virtue of similarity in chemical structure to monoaminergic neurotransmitters, MA can enter DA, NA and 5-HT axons either by passive diffusion or through DAT, NA transporter (NAT) or 5-HT transporter (5-HTT), respectively [10,12,16]. Once MA is inside the nerve terminal, it can interact with the vesicular monoamine transporter-2 (VMAT-2) to cause the release of vesicular DA, NA or less potently 5-HT into the cytoplasm [11,17] then into the extracellular space by reverse transport through DAT, NAT or 5-HTT, respectively [11,18,19]. Other molecular processes resulting in increased extracellular monoamine levels following exposure to amphetamines include the inhibition of the monoamine oxidase (MAO) enzyme [20] and the increased activity and expression of tyrosine hydroxylase (TH), the enzyme which catalyses the formation of DA from tyrosine [11,21]. 

MA-induced increases in monoamine release have been reported to be greatest for NA followed by DA and lastly 5-HT [22]. MA-induced increases in noradrenergic transmission are thought to account for its cardiovascular effects such as elevated blood pressure, cardiac arrhythmias and muscle tremor [18,23]. Whereas MA-induced increase in dopaminergic transmission is thought to underlie its addictive effects [24].

Appreciation of the widespread use of MA and its long lasting and untoward effects on the health of users and their families have led to a number of animal and human studies examining the effects of this agent on the central nervous system. This article systematically reviews findings from neuroimaging studies in adult human MA abusers, including positron emission tomography (PET), functional and structural magnetic resonance imaging (MRI) studies but excludes proton magnetic resonance spectroscopy studies of neuronal metabolites. The effects of prenatal MA exposure on children are outside the scope of this review. Although this article is not intended to systematically review molecular and cellular mechanisms of MA-induced neurotoxicity, potential mechanisms emerging from animal literature are discussed in some detail in the second section of this review. For the purposes of this review, the terms “ventral striatum” and “nucleus accumbens” are interchangeable.

## 2. MA-Induced Neurotoxicity

### 2.1. MA-Induced Monoaminergic Neurotoxicity in Animal Models

The neurotoxic effects of MA on monoaminergic neurons were first discovered in 1976, whereby two studies reported decreased brain markers of pre-synaptic DA terminals in monkeys and rats [25,26]. Kogan and colleagues reported an initial increase in striatal DA levels in the rat brain after repeated doses of MA followed by a decrease in DA levels, accompanied by decreases in striatal (40%) and nigral (45%) TH activity [25]. Concurrently, Seiden and colleagues found that long-term repeated intravenous administration of MA in increasing doses to rhesus monkeys caused a 70% reduction in caudate DA and smaller losses of NA in the midbrain (33%) and frontal cortex (52%) which lasted up to six months following the last MA injection [26]. Later studies from the same group demonstrated persistent decreases in caudate DA levels in monkeys, rats and guinea pigs, as well as a reduction in the number of DA uptake sites but no changes in NA levels in monkeys [27,28,29,30]. 

Since these initial studies, there have been numerous studies in various animal species investigating neurobiological mechanisms underlying MA-induced neurotoxicity [31]. The neurotoxic effects of MA have been demonstrated in various animal models including monkeys, guinea pigs, rats and mice and have been shown to primarily impact the dopaminergic and serotonergic systems with less prominent effects on the noradrenergic system. The effects of MA on the brains of non-human primates are thought to be the most comparable with those of humans, owing to the similarity in metabolic pathways of MA. The Rhesus monkey is a particularly useful model for studying the effects of MA, as it metabolises MA mainly via side-chain deamination, similarly to humans [32]. Guinea pigs utilise similar mechanisms of sidechain breakdown, whereas rats metabolise MA primarily by ring hydroxylation and mice utilise both pathways of sidechain breakdown and aromatic hydroxylation to an equal extent [32]. Despite the difference in MA metabolism to humans, the rat species remains the most widely used and convenient laboratory model species and studies on the effects of MA on the rat brain have resulted in valuable findings [25,27,33,34,35,36,37].

The effects of MA on the dopaminergic system have been well studied in animal models. Using PET and post-mortem analyses, Melega and colleagues reported reductions in striatal DA levels and DAT binding sites in MA-treated vervet monkeys after one week [38], three weeks [39,40] and 10–12 weeks of MA treatment [40]. Rats and mice treated with acute high doses of MA have showed long-term reductions in striatal DA levels [25,27,33,36,37,41], TH activity [35,42] and DAT binding [34,42]. Moreover, MA-treated rats exhibited reductions in VMAT-2 binding [34]. 

In addition to its neurotoxic effects on the dopaminergic system, MA has been shown to cause damage to presynaptic 5-HT terminals. Although the majority of mice strains have shown resistance to 5-HT neuron toxicity following MA administration [43,44,45], MA-induced depletion of 5-HT levels has been reported in monkeys and rats in various brain regions including the striatum [29,33,36,37,46,47], frontal cortex [29,30,36,46], hippocampus [30,37,46,47] and amygdala [36,37]. Moreover, reduced tryptophan hydroxylase activity has been reported in MA-treated rats in the striatum, hippocampus [35,47], nucleus accumbens and cerebral cortex [47]. MA has also been shown to induce decreases in 5-HTT binding in the striatum, nucleus accumbens, anterior cingulate cortex (ACC), hippocampus and amygdala [34]. 

Findings of neurotoxicity associated with the noradrenergic system in MA-treated animals have been scarce. Nonetheless, reduced concentrations of NA were observed in the frontal cortex and midbrain of rhesus monkeys [26] as well as in the striatum, cerebral cortex and hippocampus of rats following treatment with high doses of MA [48]. Subsequent studies in monkeys, rats and guinea pigs reported no changes to noradrenergic terminal markers following treatment with various dosing schedules of MA [27,29].

MA-induced monoaminergic depletion has been shown to be reversible in rats [46] and non-human primates [40], whereby partial recovery of DA synthesis and DA concentrations was observed in the striatum of vervet monkeys 10–12 weeks following treatment with MA [40]. Nevertheless, decreases in DA and 5-HT levels in the brains of rhesus monkeys have been shown to persist for at least four years following administration of high doses of MA, suggesting a potentially permanent neurotoxic effect [49]. 

MA-induced neurotoxicity in non-human primates has been thought to predominantly involve monoaminergic axonal and axon terminal abnormalities without neuronal cell death. However, neuronal cell death has been reported in rats treated with acute doses of MA resembling a binge dose pattern in the striatum [50], medial prefrontal cortex (PFC) [51] and in somatosensory cortices [52,53,54]. Moreover, loss of pyramidal neurons in the cortex and hippocampus has been observed in rats treated with escalating doses prior to a high dose challenge of MA [55]. Neuronal cell death has also been found in the striatum, frontal and parietal cortices, hippocampus and olfactory bulb of MA-treated mice [42,56,57,58,59,60] and was thought to occur via neuronal apoptosis [61,62].

### 2.2. Possible Mechanisms of MA-Induced Neurotoxicity in Animal Models

Although the exact mechanisms by which MA induces neurotoxic effects within monoaminergic systems are not fully established, several mechanisms have been suggested and discussed in animal and human literature. The involvement of DA in MA-induced neurotoxicity has been well established. As mentioned in the previous section of this article, MA can enter DA axons, due to structural similarity, either via passive diffusion or through DAT [16] and causes release of vesicular DA into the cytoplasm through interactions with VMAT-2 then out into the synaptic cleft via reverse transport [11]. Following MA-induced vesicular release, cytoplasmic DA is auto-oxidised to DA quinones, which undergo redox cycling to form superoxide radicals [63,64]. Hydrogen peroxide is produced during metabolism of DA by the MAO enzyme in the cytosol [65]. Together, superoxide radicals and hydrogen peroxide interact with transition metals to form reactive toxic hydroxyl radicals [64,66]. The resultant oxidative stress leads to the dysfunction and damage of presynaptic nerve terminal membranes [63,67]. Released DA within synaptic clefts, facilitated by MA exposure, has also been shown to have neurotoxic effects through activation of post-synaptic DA receptors [68]. This is supported by findings that treatment with DA receptor antagonists following MA exposure partially blocked the degeneration of DA nerve terminals [68]. For example, the DA D1 receptor antagonist, SCH23390, has been shown to partially inhibit the cascade of neurotoxic events following interaction of DA with the D1 receptor, by attenuating the DA-induced activation of transcription factors which have been shown to cause the death of postsynaptic neurons through apoptosis [68]. 

Monoaminergic transporters play an important role in MA neurotoxicity. As mentioned earlier, MA interacts with VMAT-2 and DAT to facilitate the release of DA into the cytoplasm of presynaptic DA terminals and synaptic clefts, respectively [11,16]. The role of DAT in MA-induced presynaptic neuronal damage has been demonstrated in DAT knockout mice which were afforded protection against MA-induced DA depletion, reactive oxygen species production and reactive astrocytosis [41]. The involvement of VMAT-2 in MA neurotoxicity has been supported by the finding that depletion of vesicular DA caused by pre-treatment with reserpine, an irreversible inhibitor of vesicular transport, enhances the toxicity of MA [69,70,71,72]. However, the exacerbated neurotoxicity reported by these studies may not be entirely attributable to the effects of MA, and may be more related to the DA depletion resulting from treatment with reserpine. The 5-HTT has also been shown to play a role in MA-induced toxicity towards 5-HT nerve terminals [34]. Various studies demonstrated attenuation of MA-induced decreases in 5-HT levels and tryptophan hydroxylase activity in the striatum following treatment with 5-HTT inhibitors such as fluoxetine, citalopram and chlorimipramine [35,73,74].

There has also been strong evidence supporting gluatamate and nitric oxide (NO) involvement in MA-induced neurotoxicity towards DA neurons [75,76]. MA has been shown to cause increases in glutamate release and activation of glutamate receptors followed by excitotoxic damage [75,76], which involves production of and interactions with NO [77]. NO interacts with superoxide radicals to form the neurotoxin preoxynitrite [78], both of which have been shown to be involved in the neurotoxic effects of MA towards monoaminergic cells [79,80].

Although a large body of evidence supports the role of DA in MA-induced cellular toxicity, it has been suggested that MA-induced DA oxidation and subsequent neurotoxicity are not necessarily preceded by increases in extracellular DA and that additional mechanisms must exist [81,82]. Under controlled temperature conditions, mice with genetically-induced DA deficiency and treated with MA still developed neurotoxicity, indicating DA-independent mechanisms must play a role in MA-induced neurotoxicity [82].

MA exposure is reported to cause temperature dysregulation; particularly, an increase in core body temperature or hyperthermia [83,84]. The hyperthermic actions of MA are thought to contribute to its neurotoxic effects, partially through potentiating MA-induced increase in free radicals and consequent oxidative stress [81,84]. For example, there was an increase in the formation of the quinone metabolites of DA during hyperthermic conditions in rats after treatment with MA, however this was blocked when MA-induced hyperthermia was prevented using low ambient temperatures [81]. Hyperthermia may contribute to MA-related dysfunction of the blood-brain barrier by mechanisms involving free radicals [84]. MA has been shown to cause breakdown of the blood-brain barrier resulting in leakage of serum albumin into the brain tissue [84]. Additionally, MA was shown to cause myelin degeneration and reactive astrocytosis in parietal and occipital cortices [84]. 

Microglia are immune cells that normally function in response to inflammation in order to protect the brain against damage and their role has been implicated in MA-induced neurodegeneration through inflammatory processes [85,86]. Activated microglia may release a variety of potentially toxic prostaglandins, cytokines and free radicals which can cause neuronal injury [85]. Moreover, MA-induced microglial activation has been reported in brain areas where neuronal degeneration was also found [34,87,88]. 

In summary, animal models of MA abuse and dependence have contributed to our understanding of the mechanisms of action of MA and its neurotoxic effects. MA causes disruption in monoaminergic transmission, particularly degeneration of DA and 5-HT terminals. Mechanisms by which MA may induce neurotoxicity include increases in oxidative stress, excitotoxicity, hyperthermia, blood-brain barrier dysfunction, reactive astrogliosis and microgliosis. Knowledge of these mechanisms can aid in the elucidation of the pathological functional and structural changes that occur in the brain of human MA abusers.

### 2.3. MA-Induced Neurotoxicity in Humans: Post-Mortem Studies

Post-mortem studies of the brains of chronic MA-abusing humans have shown reductions in presynaptic striatal DA nerve terminal markers, including DA, TH and DAT [89,90]. There were no changes observed in the levels of vesicular monoamine transporter or DOPA decarboxylase, suggesting that long-term neuronal damage in the striatum is not permanent [90]. Despite MA-induced monoaminergic depletion in post-mortem studies, chronic MA abuse was not thought to be toxic to DA neurons in the adult human brain [90]. However, post-mortem investigations suffer from the inherent limitation of studying dead human tissue samples, therefore findings from these studies may not be truly representative of the living human brain. Nevertheless, molecular targets in the live human brain can be investigated using PET.

## 3. Dopaminergic Transmission and Glucose Metabolism Changes in MA Abusers Using PET Imaging

PET is a technique which measures the radioactivity released by specific radioligands, and can therefore be used to generate cross-sectional brain images with detailed anatomical information about changes in neurotransmitter responsiveness or receptor expression [91]. PET has been widely used in drug addiction research, both as an *in vivo* measurement of various components of monoaminergic neurotransmission; particularly that of DA [92,93], and to study brain glucose metabolism, which serves a marker of brain function in many neurodegenerative diseases and psychiatric conditions including drug addiction [93,94,95]. In this section of the article, we review PET studies in human MA abusers. Study specifications and main findings are summarised in Table 1.

**Table 1:** Summary of study specifications and findings from PET studies of MA dependence.

Authors	Groups	Age (year) (Mean ± SD )	Male	MA Use Variables (Mean ± SD)	Assessed? or Regions	Radioligand(s)	Main Findings
**Iyo *et al.* 1993 [96]**	6 MA	28.0 ± 2.8	6	MA use >2 years (mean 6.9 ± 2.4 years)	D2 binding within ROIs in striatum	[^11^C]-*N*-methylspiperone (D2 and 5-HT receptor ligand)	↓ ratio of striatum: frontal cortex binding availability in MA relative to C
	10 C	28.8 ± 6.8	10	Abs > 2 months (1.5 ± 1.0 years)	5-HT2 binding within ROIs in frontal cortex		
**McCann *et al.* 1998 [97]**	6 MA	37.5 ± 8.1	3	MA use >3 years (mean 11.2 ± 7.4 years)	DAT levels within ROIs in caudate and putamen	[^11^C]WIN-35,428 (DAT ligand)	↓ DAT in caudate and putamen of MA, MC and PD relative to C
	4 MC	31.2 ± 5.6	2				
	3 PD	53.3 ± 10.4	2	Abs on average ~3 years			↓ DAT in caudate: PD > MC > MA
							↓ DAT in putamen: PD > MA > MC
	10 C	30.5 ± 10.0	4	CumMA (g) = 3437 ± 5348			
**Volkow *et al.* 2001 [94]**	15 MA	32 ± 7	6	MA use (11 ± 6 years)	D2 receptor availability within ROIs in striatum and cerebellum Regional brain glucose metabolism within ROIs in OFC, caudate and putamen	[^11^C]raclopride (D2 receptor ligand)	↓ D2 receptor availability level in striatum of MA
	20 C	31 ± 7	14	Abs > 2 weeks (5.9 ± 9.0 months)		[^18^F]FDG (glucose metabolism)	D2 receptor availability associated with metabolic rate in the orbital frontal cortex in both groups
				CumMA (g) = 12290 ± 22396			
**Volkow *et al.* 2001 [95]**	15 MA	32 ± 7	6	MA use (11 ± 6 years)	Global, regional and relative regional (normalised for global brain activity) brain glucose metabolism	[^18^F]FDG (glucose metabolism)	↑ global glucose metabolic rate in MA
							↑ regional glucose metabolic rate in parietal cortex of MA
	21 C	31 ± 8	15	Abs >2 weeks (5.9 ± 9.0 months)	Abs effects		Relative glucose metabolic rate: ↓ in thalamus, caudate, putamen and ↑ in parietal cortex of MA
					Gender effects		
				CumMA (g) = 12290 ± 22396			
							Effects persist at ≥11 months Abs
**Volkow *et al.* 2001[98]**	15 MA	32 ± 7	6	MA use (11 ± 6 years)	DAT levels within ROIs in striatum	[^11^C]*d*-*threo*-methylphenidate (DAT ligand)	↓ DAT in putamen and caudate of MA
							↓ DAT persists at ≥11 months Abs
							↓ DAT associated with motor slowing and memory impairment
	18 C	31 ± 7	12	Abs > 2 weeks (5.9 ± 9.0 months)	Abs effects		
				CumMA (g) = 12290 ± 22396			
**Volkow *et al.* 2001 [99]**	5 MA tested twice	29 ± 3	2	Early Abs: 3 ± 1.6 months	DAT levels within ROIs in striatum	[^11^C]*d*-*threo*-methylphenidate (DAT ligand)	↑ DAT in putamen and caudate of MA tested twice after protracted Abs
	5 MA tested once	35 ± 3	1	Protracted Abs > 9 months (14 ± 2 months)			
							↑ DAT in putamen and caudate of MA in protracted Abs combined (*n* = 10) *vs.* short-term abstinent MA (*n* = 12) [98]
					Neuropsychological tests of motor and memory function		
	18 C [98]	31 ± 7	12	Abs > 9 months (17 ± 10 months)			
							↓ DAT availability in short-term abstinent MA relative to C
**Sekine *et al.* 2001 [100]**	11 MA	27.4 ± 5.6	11	MA use >1 month (4.8 y ± 4.5 years)	DAT levels within ROIs in caudate/putamen, nucleus accumbens and PFC	[^11^C]WIN-35,428 (DAT ligand)	↓ DAT in caudate/putamen, nucleus accumbens and PFC of MA relative to C
	9 C	26.9 ± 4.5	9	Abs > 7 days (5.6 ± 5.7 months)	Psychiatric symptoms with the BPRS		↓ DAT in caudate/putamen and nucleus accumbens associated with MA use duration and severity on BPRS
**Sekine *et al.* 2003 [101]**	11 MA	27.4 ± 5.6	11	MA use >1 month (4.8 y ± 4.5 years)	DAT levels within ROIs in OFC, DLPFC and amygdala	[^11^C]WIN-35,428 (DAT ligand)	↓ DAT in OFC, DLPFC and amygdala in MA relative to C
							↓ DAT in OFC and DLPFC correlated with MA use duration and severity of psychiatric symptoms
	9 C	26.9 ± 4.5	9	Abs > 7 days (5.6 ± 5.7 months)	Psychiatric symptoms with the BPRS		
**Wang *et al.* 2004 [102]**	5 MA tested twice	29 ± 3	2	Early Abs: 3 ± 1.6 months	Global and regional brain glucose metabolism within ROIs in striatum, thalamus and occipital cortex	[^18^F]FDG (glucose metabolism)	↓ striatal (caudate and nucleus accumbens) glucose metabolism in MA after protracted Abs relative to C
	8 MA tested once	36 ± 3	2	Protracted Abs > 9 months (14 ± 2 months)			
							↑ thalamic but not striatal glucose metabolism in MA following protracted Abs relative to early Abs
	11 C [95]	31 ± 7	4	Abs > 9 months (17 ± 10 months)			
					Neuropsychological tests of motor function, memory function and attention		
							↑ thalamic glucose metabolism in MA associated with improved performance in motor and verbal memory tasks
**London *et al.* 2004 [103]**	*SPM:*				Global and relative regional brain glucose metabolism within ROIs in OFC, cingulate, lateral PFC, insula, amygdala, ventral and dorsal striatum and cerebellum	[^18^F]FDG (glucose metabolism)	↓ relative regional glucose metabolism in MA in ACC and insula
	17 MA	34.7 ± 1.87	11	MA use >8 years (10.1 ± 1.3 years)			
	18 C	32.3 ± 1.91	10	MA dose (g/week): 4.0 ± 0.72			↑ relative regional glucose metabolism in MA in lateral OFC, middle and posterior cingulate, amygdala, ventral striatum and cerebellum
	*Absolute glucose metabolism:*						
	14 MA	34.5 ± 2.14	10	MA use >8 years (10.2 ± 1.46 years)			Positive correlation between relative glucose metabolism in limbic regions (ACC and amygdala) and depressive symptoms
	13 C	32.6 ± 2.48	8	MA dose (g/week): 2.9 ± 0.50	Self-reports of depressive symptoms (BDI)		
**London *et al.* 2005 [104]**	17 MA	34.7 ± 1.87	11	MA use (9.29 ± 1.13 years)	Relative regional brain glucose metabolism within ROIs in OFC, cingulate gyrus, insula and hippocampus	[^18^F]FDG (glucose metabolism)	↑ error rates on CPT in MA *vs.* controls
				MA dose (g/week): 3.60 ± 0.78			
							Correlations between error rates on CPT and relative glucose metabolic rate in the cingulate (anterior and middle) and insula: negative in MA, positive in controls (cingulate only)
	16 C	33.3 ± 1.98	10	MA abusers were actively using for 30 days prior to study but were detoxified for 4–7 days prior to MRI	Auditory CPT—a vigilance test of sustained attention		
**Kim *et al.* 2005 [105]**	35 MA	35.5 ± 6.4	28	MA use: (69.78 ± 44.97 months)	Voxel-wise analysis of regional glucose metabolism in SPM 99	[^18^F]FDG (glucose metabolism)	↓ regional glucose metabolism in the right superior frontal WM in MA relative to C correlated with ↑ perseveration and nonperseveration errors on the WCST
					Gender effects		
					Smoking effects		
					WCST		Male MA *vs*. male C: results same as above
							Female MA *vs*. female C: no differences in glucose metabolism or performance on the WCST
	21 C	33.2 ± 6.4	15	Abs > 4 weeks (19.14 ± 27.20 months)			
				CumMA (g) = 0.355 ± 0.446			
**Johanson *et al.* 2006 [106]**	15 MA	32 (21–48)	11	MA use >1 year (range 1–30 years; mean 10.3 years)	DAT and VMAT-2 binding within ROIs in striatum	[^11^C]methylphenidate (DAT ligand)	↓ DAT in caudate, anterior and posterior putamen of MA
	16 C	31 (18–48)	10	Abs > 3 months (range 3 months–18 years; mean 3.4 years)	Neurocognitive tests of motor function, memory, learning, attention and executive function	[^11^C]DTBZ (VMAT-2 ligand)	↓ VMAT-2 in caudate and anterior putamen of MA
**Sekine *et al.* 2006 [107]**	12 MA	31.4 ± 6.8	7	MA use >1.5 years (6.7 ± 3.2 years)	5-HTT levels by SPM within bilateral ROIs in midbrain, thalamus, caudate, putamen, amygdala, ACC, DLPFC, OFC, temporal and cerebellar cortices	[^11^C](+)McN-5652 (5-HT transporter ligand)	↓ 5-HTT levels in the cerebral cortex (ACC, temporal, OFC, DLPFC), midbrain, thalamus, caudate, putamen and cerebellum of MA
	12 C	31.8 ± 6.6	7	Abs > 0.5 year (1.6 ± 1.3 years)			Negative correlation between 5-HTT levels in the OFC, ACC and temporal areas and levels of aggression amongst MA
					AQ		
**Berman *et al.* 2008 [108]**	10 MA tested at two time points	33.1 ± 6.79	9	MA use: (8.20 ± 4.5 years)	Absolute brain glucose metabolic rate (both global and regional)	[^18^F]FDG (glucose metabolism)	↑ global glucose metabolic rate in MA after 4 weeks of Abs
				Abs 1: 5–9 days (6.7 ± 1.6 days)			↑ regional glucose metabolism in cortex (maximally in parietal regions) after 4 weeks of Abs
				Abs 2: 4 weeks after time point 1 (27.6 ± 0.96 days)			
							↑ metabolic rate in parietal regions correlated with improved reaction times on the auditory vigilance task
					Regional relative radioactivity		
	12 C	33.7 ± 7.49	7				
				MA dose (g/week): 2.01 ± 2.0			
					Auditory vigilance test of sustained attention		
							No change in metabolic rate in subcortical areas after 4 weeks of Abs
**Boileau *et al.* 2008 [109]**	16 MA	27.8 ± 5.7	11	MA use >2 years (5.1 ± 3 years)	VMAT-2 binding within ROIs in the striatum, thalamus and midbrain	[^11^C]DTBZ (VMAT-2 ligand)	↑ VMAT-2 binding in caudate, putamen and ventral striatum of MA
					Voxelwise analysis of VMAT-2 binding over the striatum, globus pallidus, thalamus and midbrain		
	14 C	29.7 ± 5.4	11	Abs > 1.5 days (19 ± 24 days)			↑ VMAT-2 binding in caudate negatively correlated with Abs duration
					Neuropsychological and cognitive testing		
**Lee *et al.* 2009 [110]**	22 MA	35.6 ± 8.4	13	MA use >(14.6 ± 8.2 years)	D2/D3 receptor binding within ROIs in the caudate, putamen and nucleus accumbens	[^18^F]fallypride (D2/D3 receptor ligand)	↓ D2/D3 receptor availability in MA in caudate, putamen and nucleus accumbens
				MA dose (g/week): 3.0 ± 3.2			
	30 C	34.9 ± 8.9	16		BIS		↓ D2/D3 receptor availability in MA in caudate and nucleus accumbens correlated with higher impulsivity
				Abs 4–10 days			
**Boileau *et al.* 2010 [111]**	9 C before and 2 ↑ after AMPH (0.4 mg/kg)	26 ± 6	6	N/A	VMAT-2 binding within ROIs in the caudate, putamen, ventral striatum and substantia nigra	[^11^C]DTBZ (VMAT-2 ligand)	No ↑ in VMAT-2 binding following AMPH dose
							Slight 5% ↓ in VMAT-2 binding following AMPH dose
**Wang *et al.* 2011 [112]**	16 MA tested twice	39.2 ± 4.9	13	MA use (13.1 ± 7.2 years)	D2 receptor availability within ROIs in dorsal striatum, ventral striatum and cerebellum, over 2 scanning sessions, following placebo and methylphenidate (60 mg)	[^11^C]raclopride (D2 receptor ligand)	↓ D2 receptor availability at baseline in caudate of MA
							↓ striatal D2 receptor availability across both groups after methylphenidate (smaller ↓ in MA in the left putamen)
				Abs 1: >2 weeks (within 6 months of last MA use)			
				Abs 2: 9 months after first PET scan at time point 1			
							Non-relapsing MA (*n* = 10) showed D2 receptor changes after methylphenidate similar to that of controls
	15 C	37.2 ± 4.3	13				
					Voxelwise analysis of D2 receptor availability		
				MA dose (g/day): 1.2 ± 1.0			
							Relapsing MA (*n* = 6) had ↓ D2 receptor availability in dorsal striatum, no D2 receptor change after methylphenidate
**Boileau *et al.* 2012 [113]**	16 MA	27.93 ± 5.66	12	MA use >2 years (5.1 ± 2.7 years)	D3 receptor availability within ROIs in the striatum and substantia nigra	[^11^C]-(+)-PHNO (D3 receptor-preferring ligand)	↑ [^11^C]-(+)-PHNO binding in MA in substantia nigra, globus pallidus (NS) and ventral striatum (NS)
				Abs > 6 days (18.5 ± 20.5 days)			
	16 C	28.43 ± 5.01	14		Voxelwise analysis of D3 receptor availability		↓ [^11^C]-(+)-PHNO binding in MA in dorsal striatum
				MA dose (mg/dose): 325 ± 167			

5-HTT: serotonin transporter; [^11^C]DTBZ: [^11^C]dihydrotetrabenazine; [^11^C](+)McN-5652: *trans*-1,2,3,5,6,10-β-hexahydro-6-[4-(methylthio)phenyl]pyrrolo-[2,1-*a*]isoquinoline; [^11^C]-(+)-PHNO: [^11^C]-(+)-propyl-hexahydro-naphtho-oxazin; [^18^F]FDG: [^18^F]fluorodeoxyglucose; Abs: abstinence duration; ACC: anterior cingulate cortex; AMPH: amphetamine; AQ: aggression questionnaire; BDI: Beck Depression Inventory; BIS-11: Barratt Impulsiveness Scale; BPRS: Brief Psychiatric Rating Scale; C: control group; CPT: continuing performance task; CumMA (g): lifetime cumulative methamphetamine dose in grams; DAT: dopamine transporter; DLPFC: dorsolateral prefrontal cortex; MA: methamphetamine group; MC: methcathinone group; MRI: magnetic resonance imaging; N/A: not applicable; NS: not significant; OFC: orbitofrontal cortex; PD: early Parkinson’s disease group; PFC: prefrontal cortex; ROI: region of interest; SPM: statistical parametric mapping; VMAT-2: vesicular monoamine transporter-2; WCST: Wisconsin card sorting test; WM: white matter.

### 3.1. PET Studies of Postsynaptic DA Receptors in MA Abusers

DA D2 receptor availability has been used as a biomarker for the investigation of postsynaptic DA neuron function in MA abusers. The most commonly used radioligand for this purpose has been [^11^C]raclopride, a reversible D2 receptor radioligand [93]. [^11^C]raclopride has been well validated for its accuracy and precision of D2 receptor measurement in the human ventral striatum, using the endogenous competition technique [114]. Volkow and colleagues have conducted a series of PET studies comparing abstinent MA abusers with a group of subjects with no history of drug abuse [94,95,98,99,102,112]. They investigated postsynaptic D2 receptor availability using PET with [^11^C]raclopride and found a lower level of postsynaptic striatal D2 receptor availability in the caudate (16%) and putamen (10%) of MA abusers relative to the comparison group [94]. The reduced D2 receptor availability was associated with reduced glucose metabolic rate in the orbitofrontal cortex (OFC), as measured by PET with [^18^F]fluorodeoxyglucose (FDG) [94]. This association has also been observed in cocaine abusers [115] and suggests that dysregulation of the OFC in drug addicted individuals may be mediated by D2 receptors, and could be one of the mechanisms underlying compulsive drug-taking behaviour [94].

A more recent longitudinal study assessed the extent to which DA function in the striatum of MA abusers might predict recovery [112]. D2 receptor availability was measured using PET and [^11^C]raclopride in MA abusers and healthy controls following the administration of a 60 mg oral dose of methylphenidate or placebo [112]. Methylphenidate is a DAT inhibitor [93], and was used to enable a comparable measurement of DA release in MA abusers *vs*. controls [112]. Testing in MA abusers was conducted twice, the first time within six months of last MA use, and the second time nine months after; controls were tested at corresponding time points [112]. During early withdrawal, MA abusers had lower D2 receptor availability than controls in the caudate [112]. As expected, following the methylphenidate challenge there was a decrease in striatal (caudate, putamen, ventral striatum) D2 receptor availability in both MA abusers and controls [112]. However, the decrease was smaller in MA abusers than controls in the left putamen possibly reflecting both decreased DA release as well as a reduction in D2 receptor levels in the striatum of MA abusers, as has also been observed with cocaine abuse [116]. Interestingly, the MA abusers who had lower D2 receptor levels at baseline than controls in the dorsal striatum (caudate and putamen), also experienced no D2 receptor alterations following methylphenidate challenge and relapsed during the nine month follow-up period [112]. Correspondingly, MA abusers who experienced a DA increase post-methylphenidate, comparable with that of controls, did not relapse and completed a nine month abstinence period [112]. The authors concluded that abstinent MA abusers with low striatal DA function may be less sensitive to natural reinforcers, which may maintain their reward-directed drug seeking behaviour, making them more prone to relapse [112]. 

PET imaging was used in an earlier study of MA-induced psychosis in long-term MA abusers who had been abstinent for at least 2 months [96]. The radioligand ^11^C-N-methylspiperone was used to assess the role of D2 and 5-HT2 receptors in the susceptibility to MA-induced psychosis [96]. Although there were no group differences in binding availability, MA abusers were found to have a significantly decreased ratio of binding availability of D2 receptors in the striatum relative to 5-HT2 receptors in the frontal cortex [96]. Since DA release can be regulated by 5-HT2 receptors in the frontal cortex [117], it was suggested that the relative increase in 5-HT2 receptor density and decrease in D2 receptor density might be the result of a long-term dysfunction of DA release by DA neurons in chronic MA abusers [96].

Dopaminergic dysfunction associated with MA use and drug addiction has mainly been reported as a reduction in the levels of DA and D2 receptor densities [90,94,110,112]. These findings support the hypothesis that MA addiction should be treated with agents that increase DA signaling, albeit none have been successful enough to be approved for this use [118]. More recently, addiction research using PET has turned its attention to the D2-like D3 receptor subtype, which is highly expressed in limbic areas associated with reward and motivation such as the nucleus accumbens [119,120]. In a PET study using [^18^F]fallypride, a radioligand with affinity for D2-like D3 receptors, MA abusers were shown to have lower D2-like D3 receptor availability than healthy controls in the caudate (16.1%), putamen (12.6%) and nucleus accumbens (8.4%) [110]. In the MA abusers, lower availability of D2/D3 receptors in the caudate and nucleus accumbens was associated with higher impulsivity as measured by the Barratt Impulsiveness Scale [110]. Both animal and postmortem human studies suggest D3 receptor levels are pathologically increased in cocaine addiction [121,122,123,124]. If the same applies to MA addiction, then treatment with a dopaminergic agent may be inappropriate as it may remedy a D2 receptor deficit but increase an already high level of D3 receptors [113]. 

Boileau and colleagues tested the hypothesis that D3 receptor availability is elevated in MA abusers using PET with [^11^C]-(+)-propyl-hexahydro-naphtho-oxazin ([^11^C]-(+)-PHNO), a D3-preferring ligand [113] and found that D3 receptor availability was 46% higher in the substantia nigra. The MA group also exhibited lower [^11^C]-(+)-PHNO binding in the D2-rich dorsal striatum [113]. The authors concluded that D3 receptors are upregulated in the brain of MA abusers and speculated whether selective antagonism of D3 receptors would lead to a reduction in drug taking behaviour [113]. This study was the first *in vivo* neuroimaging study of D3 receptor density in drug addiction, though there were some caveats. For example, the sample of MA abusers were polydrug users and also used cocaine, MDMA, benzodiazepines, opiates, THC and ketamine (80%, 53.3%, 33%, 46%, 56.2% and 25% of the MA group, respectively) [113]. Despite MA being their primary drug of choice (taken by all users), the results may not be specific to MA, as other recreational drugs also affect DA receptor density and may confound the results [113]. Further, while [^11^C]-(+)-PHNO is a useful radioligand for the study of D3 receptors owing to its higher differential binding at this receptor subtype [125] it, in common with all dopaminergic radioligands, lacks absolute specificity for a particular receptor subtype rendering inference from binding results where regions contain both D2 and D3 receptor subtypes challenging [113]. 

### 3.2. PET Studies of Brain Glucose Metabolism in MA Abusers

Brain glucose metabolism has been widely studied in drug addiction as an indicator of brain function [93,94,95]. Volkow and colleagues’ group of MA abusers underwent detailed glucose metabolic studies with PET following the administration of [^18^F]FDG and were found to have 14% greater global (whole brain) glucose metabolism than their matched controls [95]. This was unexpected since other drugs of abuse such as alcohol and cocaine reportedly caused hypometabolism [93]. Because MA abusers were previously reported in a proton magnetic resonance spectroscopy study to have increased levels of myo-inositol, a glial marker [126], the authors speculated that the global hypermetabolism observed in MA abusers could reflect gliosis or inflammatory processes [95]. Regional brain glucose metabolism was increased in MA abusers relative to controls in all brain cortices, however only the metabolic increase in the parietal cortex (20%) was significant [95]. Following normalisation for global activity, relative glucose metabolism was higher in the parietal cortex (5%) but lower in the striatum (caudate, 12% and putamen, 6%) and thalamus (17%) of MA abusers [95]. All effects persisted at ≥11 months abstinence, although this was inferred from a separate analysis of a very small sample of 3/15 MA abusers who remained abstinent for ≥11 months. The metabolic decrease in the striatum, which receives direct input from DA cells, and the thalamus, which mainly receives dopaminergic input from the striatum [127] was thought to reflect MA-induced neurotoxicity in DA cells [29,128,129] and disruption of DA-linked pathways [95]. Since the parietal cortex is poorly innervated by DA terminals, it was concluded that MA caused metabolic abnormalities in regions innervated by DA cells (directly in the striatum, indirectly in the thalamus) and those innervated by other neurons (parietal cortex) [95]. 

Of the original subjects reported by Volkow and colleagues [95], five MA abusers remained abstinent for nine months or longer following their first PET scan and underwent a second PET scan at this time point [102]. Striatal metabolism did not recover, suggesting long-lasting changes in dopaminergic neurons [102,130], or decreased cortical input to the striatum resulting from MA-induced excitotoxicity in cortical cells [131]. Interestingly, relative regional metabolism in the thalamus increased following protracted (12–17 months) abstinence relative to early (3 ± 1.6 months) abstinence [102]. The increased thalamic metabolism was functionally significant as it was associated with improved performance in motor and verbal memory tasks [102]. The thalamus receives indirect dopaminergic input from the striatum via gamma-aminobutyric acid (GABA) cells. Hence, the recovery of thalamic metabolic function after protracted abstinence from MA may reflect a neuroadaptive response by GABA cells to compensate for dopaminergic deficits [102]. However, a larger sample size of long-term abstinent MA abusers and controls may have been required to improve the generalizability of these findings.

Another study reported reduced regional glucose metabolism in the right superior frontal white matter of MA abusers who had been abstinent for an average of 1.5 years [105]. The frontal hypometabolism was associated with a larger number of total errors, perseveration errors and nonperseveration errors during the Wisconsin card sorting test (WCST) [105]. Poor performance during the WCST indicates deficits in decision-making and frontal executive function, hence this result was in accord with prior studies reporting failure of MA abusers to activate their PFC during decision-making [7,132]. In a sub-analysis of gender differences, the same findings of frontal hypometabolism and impaired frontal executive function were found in male MA, but not female MA abusers [105], possibly due to estrogen’s neuroprotective effects [133]. 

London and colleagues studied regional cerebral glucose metabolism in relation to mood disturbances in a group of recently abstinent (4–7 days) MA abusers [103]. They reported metabolic abnormalities in limbic and paralimbic cortices, including decreased relative regional glucose metabolism in MA abusers in the ACC and insula [103]. There was an opposing hypermetabolism in the lateral OFC, middle and posterior cingulate, amygdala, ventral striatum and cerebellar vermis [103] which might reflect the short abstinence period since these regions have been previously implicated in drug craving [134,135,136,137,138,139]. Robust hyperactivity was found in the ventral striatum and hypoactivity in the ACC of MA abusers [103], both regions being heavily innervated with DA [140]. Thus, glucose hypometabolism in the ACC may reflect dopaminergic deficits in this region [103]. 

Reduced glucose metabolism in the ACC was studied in relation to affect [103] and sustained attention [104] in the same subjects. MA abusers made more errors than comparison subjects during a vigilance test of sustained attention [104]. Higher error rates of MA abusers were associated with lower glucose metabolism in the ACC and insula, suggesting these regions are involved in deficits of sustained attention [104]. There was a positive correlation between severity of depressive symptoms, as measured by the Beck Depression Inventory (BDI) and relative glucose metabolism in the ACC and amygdala which have been linked with negative affect [103]. 

Berman and colleagues [108] compared glucose metabolism in the brain of MA abusers between very early (less than 1 week) [103,104] and short-term (average of 3 months) [95] abstinence periods. PET with [^18^F]FDG was used 5–9 days after abstinence and then following an additional four weeks in MA abusers; a group of healthy controls were tested at corresponding times [108]. After four weeks of abstinence, MA abusers showed a 10.9% greater global metabolic rate and higher regional glucose metabolism in cortical regions with no change in subcortical regions [108]. Specifically, there was a 20% increase in the metabolic rate in the parietal cortex of MA abusers which correlated with improved reaction times on an auditory vigilance task, suggesting improved vigilance and sustained attention after the first week of abstinence [108]. 

In summary, glucose metabolism has been studied in the brain of MA abusers during very early abstinence (4–7 days), and was found to be lower in the ACC and insula and higher in the ventral striatum [103,104]. During short-term abstinence (mean 3 ± 1.6 months), relative glucose metabolism was higher in the parietal cortex and lower in the striatum and thalamus [95]. During their first year of abstinence MA abusers continued to have metabolic deficits in the striatum, however their thalamic metabolism normalised [102]. During the first month of abstinence, a critical period when relapse often occurs, there were no metabolic changes in subcortical regions but a widespread increase in cortical glucose metabolism with a maximal increase in the parietal cortex [108]. However, this cortical hypermetabolism caused an increase in whole brain metabolism, and would lead to an increase in any relative regional measure scaled to the global mean [108]. Therefore, relative metabolic measures in the absence of absolute measures must be interpreted with caution [108].

Although some studies report glucose metabolic differences in MA abusers in similar anatomical locations, others report non-coincidental locations. Discrepancies in the anatomical locations of metabolic disturbances in the brain of MA abusers may be due to variable differences in MA use *i.e.*, duration of use, route of administration (intravenous *vs*. smoked), cumulative dose, duration of abstinence and exposure to other drugs. In saying that, it is possible that brain areas reported to have glucose metabolic deficits in different studies may have functional connections to each other, for example, Volkow and colleagues reported an association between striatal D2 receptor availability and glucose metabolism in the OFC of MA abusers [94].

### 3.3. PET Studies of Presynaptic Monoamine Nerve Terminals in MA Abusers

Presynaptic DA cell terminals have been studied in MA abusers by several research groups. Volkow and colleagues investigated DA terminal density in the abstinent MA abusers, using PET with [^11^C]*d*-*threo*-methylphenidate, a DAT ligand [98]. The group of MA abusers had significantly lower DAT levels in the striatum with a mean difference of 27.8% in the caudate and 21.1% in the putamen [98]. Similar findings were reported in an earlier PET study using [^11^C]WIN-35,428, a DAT ligand, including reductions in DAT levels of 23% and 25% respectively in the caudate and putamen of MA abusers who had been abstinent for 3 years on average, relative to controls [97]. A more recent PET study reported a 15% decrease in striatal DAT levels in long-term abstinent MA abusers [106]. The decrease in DAT levels in the striatum of MA abusers has been associated with functional impairments including motor slowing and memory deficits [98]. Apart from the striatum, abstinent MA abusers have also been found to have significantly lower DAT levels in the nucleus accumbens, anterior PFC, OFC, dorsolateral PFC (DLPFC) and amygdala [100,101]. Reductions in DAT in the caudate/putamen, nucleus accumbens, OFC and DLPFC were associated with duration of MA use and severity of psychiatric symptoms [100,101].

It was suggested that reduction in DAT levels may be long lasting as it persisted in those who were abstinent for longer than 11 months in one study [98], and three years in another [97]. However, a longitudinal PET study by Volkow and colleagues using [^11^C]*d*-*threo*-methylphenidate in five MA abusers during short-term (<6 months) and long-term (12–17 months) abstinence, reported increases in DAT levels of 19% and 16% in the caudate and putamen, respectively [99]. The extent of DAT recovery was associated with severity of abuse and the duration of abstinence [99]. Interestingly, performance on neuropsychological tests of motor and memory function did not improve from short- to long-term abstinence, suggesting incomplete functional recovery [99]. Although it is difficult to draw robust conclusions from findings of Volkow and colleagues’ longitudinal study [99] due to the small sample size, several ideas have been discussed. It is possible that DAT loss may reflect a neuroadapative process, whereby DAT expression is down-regulated to compensate for DA depletion in the addicted brain [97,98,99], as has been shown following amphetamine abuse [15]. However, it is also possible that reduced DAT levels reflects a neurotoxic effect whereby MA causes DA nerve terminal degeneration [97], which may [99] or may not [97,98] recover following prolonged abstinence. Alternatively, following MA-induced damage to DA terminals, the remaining viable terminals may increase their synaptic arborisation which could account for the observed increase in DAT with protracted abstinence [99]. Longitudinal studies with larger sample sizes are warranted to gain further understanding into the association between DAT levels and abstinence status and duration in MA abusers. 

Another biomarker of DA neuron terminal integrity is VMAT-2 which is responsible for the redistribution of monoamines such as DA from synaptic vesicles to the cytosol [4]. The first study of VMAT-2 densities was a post-mortem study of human MA abusers which reported no change in VMAT-2 levels despite significant reductions of DA and DAT levels, and thus suggested MA may not be toxic to DA neurons [90]. This study, however, suffered from a small sample size and from the uncertainty of whether post-mortem findings would generalise to the living human brain. VMAT-2 densities were then studied in abstinent MA abusers using PET with dihydrotetrabenazine ([^3^H]DTBZ) and found to be only 10% lower than comparison subjects [106]. The lack of substantial changes in VMAT-2 levels in this study may be attributed to the wide range of abstinence duration amongst the sample of MA abusers (range 3 months–10 years, mean 3 years). Despite the lack of correlation between VMAT-2 levels and duration of abstinence [106], the wide duration of abstinence range may have resulted in a wash-out of significant effects. Alternatively, neuronal recovery may have occurred following prolonged abstinence in some of the subjects [99,100]. Nonetheless, the modest but significant reduction in VMAT-2 availability in MA abusers reported by this study, along with its lack of correlation with duration of abstinence may reflect permanent structural changes [106]. However, the results of this study may have been confounded by the use of drugs other than MA such as alcohol, cocaine, opiates and marijuana [106]. 

In a recent study by Boileau and colleagues investigating VMAT-2 levels in MA abusers during early abstinence using PET and (+)[^11^C]DTBZ [109], striatal binding was increased by 22% in the caudate, 12% in the putamen and 11% in the ventral striatum during early abstinence [109]. In the caudate, higher (+)[^11^C]DTBZ binding levels were associated with shorter abstinence [108]. The increase in (+)[^11^C]DTBZ binding in recently abstinent MA abusers suggests reduced competition with DA at the VMAT-2 receptors and further verifies the low levels of stored striatal DA in MA abusers [89,90]. However, this finding was not supported by a more recent study by Boileau and colleagues’ in non-drug users administered a low acute oral dose (0.4mg/kg) of amphetamine [111]. Non-drug users are likely to have normal levels of striatal DA, and a low dose (mean ± SD 32.2 ± 5.1 mg) of amphetamine is unlikely to induce sufficient DA depletion to result in increased (+)[^11^C]DTBZ binding [111].

5-HT transporter density was compared in a group of abstinent MA abusers and matched controls using PET with *trans*-1,2,3,5,6,10-β-hexahydro-6-[4-(methylthio)phenyl]pyrrolo-[2,1-*a*]isoquinoline ([^11^C] (+)McN-5652), a 5-HT transporter ligand [107]. 5-HT transporter density levels were significantly lower in the cerebral cortex (ACC, OFC, DLPFC, temporal regions), midbrain, thalamus, caudate, putamen and cerebellum of abstinent MA abusers [107]. The lower 5-HT transporter levels in the OFC, ACC and temporal regions were associated with higher levels of aggression amongst MA abusers, as measured by the Aggression Questionnaire [107]. 

In summary, addiction research utilising PET has made significant contributions to the field and has several advantages. Selective labeling of PET radioligands can be controlled to yield highly selective receptor specificities [93]. The sensitivity of PET is to sub-nanomolar concentrations, in comparison with the sensitivity of MRI which is in the millimolar range [93]. However, in comparison with MRI, PET imaging suffers from poor temporal (45 s *vs.* 1–3 s) and spatial (4 mm *vs*. 1 mm) resolution [93]. All PET studies discussed in this review, with the exception of two by London and colleagues [103,104], utilised abstinent MA abusers. This highlights a lack of PET studies comparing dopaminergic function and glucose metabolism in active *versus* abstinent MA abusers. There is also an ongoing need for longitudinal studies of MA addiction, to settle the continuing debate about whether drug seeking and dependence cause functional deficits or that reported deficits could have predated and predisposed individuals to drug use. The longitudinal studies [99,102] discussed herein also have very small sample sizes (e.g., *n* = 5) which limits the applicability of their findings. Additionally, the lack of a comparison group by some studies [99,102,112], at the second time point corresponding with protracted abstinence fails to allow for time-related changes to be properly accounted for.

## 4. Functional and Cognitive Deficits in MA Abusers Using Functional MRI (fMRI)

Functional MRI (fMRI) is a relatively new technique which emerged 20 years ago [141], and has been increasingly utilized since then with the number of published articles rising to a peak during the year 2010 [142]. fMRI utilises the magnetic properties of deoxygenated and oxygenated blood to form a blood-oxygen-level-dependent (BOLD) signal [143]. Blood deoxygenated haemoglobin (deoxyhaemoglobin) is paramagnetic and can induce a change in the magnetic field, producing contrast in images obtained from MRI [143]. fMRI has been frequently used to study brain activity in awake humans during rest or whilst performing cognitive, emotional or motor tasks [144]. 

Sensory or cognitive challenge-related neuronal firing initially causes an increase in oxygen demand/usage in the brain [145]. However, this is shortly (within a few seconds) followed by a larger increase in blood flow and blood volume. This results in greater oxygen demand coupled with a net reduction in paramagnetic deoxyhaemoglobin levels leading to a change in magnetic field, detected as MRI contrast or the BOLD signal [146,147]. The haemodynamic response measured by fMRI takes 4–5 s to peak and a total of 15–20 s to rise and fall, thus it is a relatively slow signal [147]. Although special measures can be undertaken during fMRI data analysis to account for it, the relatively poor temporal resolution remains an inherent limiting factor to all fMRI studies. Because the BOLD signal is not an absolute measure, the lack of an ‘implicit baseline’ is a consideration that must be addressed during experimental design [144].

The fMRI technique has several advantages over other scanning techniques including its excellent spatial resolution. For instance, in comparison with electroencephalography (EEG) which can detect electrical activity only at the surface of the brain, fMRI can detect magnetic signal from deeper structures including subcortical nuclei [142]. Moreover, a much higher spatial resolution can be obtained using fMRI (1–3 mm) than can be obtained with PET (4 mm) [93]. Therefore, fMRI enables examination of human brains, healthy or affected by disease, during performance of cognitive tasks and is an ideal technique for studying long-term changes owing to its non-invasive and safe nature [144]. 

There have been several fMRI studies in drug dependence but relatively few in MA abusers. This section of the article reviews fMRI studies in MA dependence in relation to decision-making, reward processing and cognitive control. A summary of study specifications and findings is presented in Table 2. Studies of emotion regulation are beyond the scope of this review. In this section of the review, the term “activation(s)” refers to clusters of statistically significant within- or between-subject BOLD signal differences as measured by fMRI.

**Table 2:** Summary of study specifications and findings from fMRI studies of MA dependence.

Authors	Groups	Age (year) (Mean ± SD)	Male	MA Use Variables (Mean ± SD)	Tesla (T)	Task	Behavioural Measures and Regions on fMRI	Main Findings
**Paulus *et al.* 2002 [7]**	10 MA	41.1 ± 2.4	10	MA use >12 years (mean 19.6 ± 6.9 years)	1.5	Decision-making tasks:	Task: Response bias, latency and mutual information measures	Two-choice prediction task: MA ↑ win-stay/lose-shift responses relative to C and number of responses influenced by immediately preceding outcomes negatively correlated with Abs
							fMRI: Whole-brain voxel-wise fMRI + ROI regressions with MA use characteristics	
								Two-choice prediction task *vs*. two-choice response task: Activation of prefrontal and parietal cortices in both MA and C.
						Two-choice prediction task		
	10 C	42.3 ± 1.9	10	Abs > 6 days (mean 22.4 ± 3.5 days)				
						Two-choice response task		
								↓ DLPFC activation and no ventromedial cortex activation in MA
							Abs effects	
**Paulus *et al.* 2003 [148]**	14 MA	41.1 ± 2.0	14	MA use >4 years (mean 16.8 ± 2.6 years)	1.5	Decision-making tasks:	Task: Response bias, latency and mutual information measures	Group effect: MA ↑ win-stay/lose-shift responses and ↓ activation of bilateral inferior PFC, DLPFC, bilateral parietal cortex, L post-central gyrus, and L STG relative to C
						Two-choice prediction task (3 success rates)		
							fMRI: Whole-brain voxel-wise fMRI analysis + ROI regressions with MA use characteristics	Error-rate effect: Both MA and C: Activation of R insula, R IFG, R MFG and L MFG. Lower error rates correlated with ↑ activation in both groups
								Group by error-rate effect: ↑ activation in: L medial frontal gyrus, insula, precuneus and inferior parietal lobule in C during low error rates and in MA during the most unpredictable condition
	14 C	38.7 ± 1.7	10	Abs > 6 days (mean 25.0 ± 2.7 days)				
						Two-choice response task		
							Abs effects	
								Correlations: Negative correlation between MA use duration and activation in L MFG, ACC and L precuneus. Positive correlation between Abs duration and activation in L medial frontal gyrus
**Paulus *et al.* 2005 [149]**	22 non-relapsing MA	40.3 ± 8.8	22	MA use: 14.9 ± 10 years	1.5	Decision-making tasks:	fMRI: Whole-brain voxel-wise fMRI analyses: 2 exploratory analyses, a step-wise linear discriminant function analysis and a step-wise Cox regression analysis	Two-choice prediction task *vs*. two-choice response task: ↑ bilateral PFC, striatum, PPC and anterior insula
				Abs: 27.4 ± 8.3 days				Relapsing *vs.* nonrelapsing MA: ↓ activation in PFC, insula and parietal cortex
						Two-choice prediction task		
				Follow-up: 437 ± 165 days				Step-wise discriminant function analysis results: R insula, R PCC, R MTG best predicted relapsing (17/18) and non-relapsing (20/22) MA
	18 relapsing MA	41.9 ± 9.0	18	MA use: 17.3 ± 8 years				
						Two-choice response task		
				Abs: 27.8 ± 11 days				Step-wise Cox regression analysis results: R MFG, R MTG and R PCC best predicted time to relapse
				Follow-up: 440 ± 304 days				
**Monterosso *et al* . 2007 [150]**	10 MA	33.8 ± 8.1	8	MA use: 7.7 ± 8.9 years	3	Delay discounting tasks:	Task: delay discounting rate index for each subject	Behavioural results (pre-scanner task): ↑ delay discounting in MA relative to C
						Outside scanner		
				MA dose (g/week): 4.8 ± 7.9				
							fMRI: Whole-brain voxel-wise fMRI analyses: Between-group comparisons, correlational analyses between degree of delay discounting and task-related activation change	fMRI results—combined groups: “Hard choice > no choice” contrast: ↑ activation in VLPFC, DLPFC, dorsal ACC and IPS. “Hard choice > easy choice” contrast: ↑ activation in R VLPFC and ACC/SMA
	13 C	29.7 ± 7.2	12	MA actively using for 30 days prior to study, detoxified for 5–7 days prior to study				
						Paired with fMRI (3 conditions: “hard choice”, “easy choice” and “no choice”)		
								fMRI results—MA *vs*. C: “Easy choice *vs*. hard choice” contrast: ↓ recruitment in C but not in MA
								Correlational analysis results: No relationship between task-related activation in frontoparietal circuit and higher delay discounting in MA
**Hoffman *et al.* 2008 [151]**	19 MA	34.8 ± 10.0	13	MA use: 5.1 ± 5.0 years	3	Delay discounting task paired with fMRI [conditions: immediate choice (easy and hard), delayed choice (easy and hard), magnitude estimation (control)]	Task: delay discounting rate index for each subject	Behavioural results: ↑ delay discounting in MA relative to C
								fMRI results—combined groups: “Hard choice > magnitude estimation”: ↑ activation in bilateral dorsal ACC, medial SFG, PCC, V/DLPFC, right insula and PPC including IPS
							fMRI: Whole-brain voxel-wise fMRI analyses: Regression analyses for between-group comparisons including the index of delay discounting rate as a covariate	
				Abs: 48 ± 17 days				
	17 C	36.7 ± 9.9	12					fMRI results—MA *vs*. C: “Hard choice > magnitude estimation” in controls > MA: ↑ activation in bilateral precuneus, right caudate, right ACC and right DLPFC. Discounting magnitude correlated positively with the difference in activation in the right amygdala, SFG, PCC and PPC
				MA dose (g/day): 1.2 ± 1.1				
**Leland *et al.* 2008 [152]**	19 MA	40.4 ± 9.9	17	MA use >4 years (mean 17.4 ± 10.0 years)	1.5	Go/nogo response inhibition task (including “go, cue”, “go, noncue”, “nogo” stimuli)	Task: RT, hit rate, false alarm rate, beta and d-prime	Behavioural results: ↓ false alarm rate in MA
								fMRI results: Inhibition contrast: ↑ inhibition-related activation in ventral ACC in both MA and C separately
								fMRI results: Cueing contrast: Between-group analysis: ↑ cueing-related activation in MA *vs*. C in ventral and dorsal ACC. Separate group analyses: ↑ cueing-related activation in ventral and dorsal ACC in MA, no change in C
							fMRI: Whole-brain voxel wise fMRI analysis + ROI (bilateral dorsal ACC and bilateral ventral ACC) analyses with 2 contrasts: inhibition (nogo-go) and cueing (cue-noncue)	
	19 C	40.3 ± 8.1	16	Abs > 25 days (mean 33.9 ± 5.9 days)				
								fMRI results: Regression of false alarm rates and cueing-related activation: Cueing-related activation in the ventral ACC negatively correlated with false alarm difference score
**Salo *et al.* 2009 [153]**	12 MA	35.7 ± 7.7	5	MA use >5 years(mean 13.9 ± 5.7 years)	3	Modified Stroop colour-word task with 2 conditions: congruent and incongruent	Task: RT of Stroop conflict effect, error rates, trial-to-trial RT adjustment	Behavioural results: No group difference in Stroop conflict effect or accuracy rates. Across groups: ↑ error rate during incongruent > congruent. ↓ RT adjustment effect in MA relative to C
	16 C	30.2 ± 8.9	8	Abs > 2 months (mean 4.1 ± 2.8 months)			fMRI: ROIs in ACC and PFC with 2 contrasts: Stroop conflict effect and trial-to-trial adjustment effects	
								fMRI results: ↑ activation of ACC in both groups during incongruent trials. ↓ activation of R PFC in MA relative to C corresponding with ↓ trial-to-trial adjustment
**Nestor *et al.* 2011 [154]**	10 MA	33.5 ± 9.3	5	MA use: 8.3 ± 3.7 years	3	Stroop colour-word task—3 conditions: congruent, incongruent and rest	Task: RT and accuracy (% errors) for each condition and Stroop effect	Behavioural results: Group effects: ↑ % errors and ↑ RT in MA relative to C across both conditions. Condition effect: ↑ RT in incongruent relative to congruent condition across both groups. GroupxCondition effect: ↑ RT in MA relative to C during incongruent and congruent conditions
								fMRI results—Analysis 1: Stroop effect contrast: ↓ activation in L cerebellum and ↑ activation in PCC, R precuneus, bilateral occipital poles, ICC, PPG, bilateral ACC, R paracingulate cortex, R frontal pole, L medial frontal cortex
							fMRI: Voxel wise whole-brain fMRI analysis—2 analyses: 1. Without RT covariate and 2. Including RT covariate, 3 contrasts: congruent, incongruent and Stroop effect	
	18 C	36.4 ± 10.4	11	Abs: 4–7 days				
				MA dose (g/week): 8.4 ± 7.3				
								fMRI results—Analysis 2: Incongruent contrast): ↓ activation in R IFG, R supramarginal gyrus, R PCG, R SMA/ACC, R occipital pole, R LOC, R ITG, R TOFC, R MTG, R FOC, R temporal pole and anterior insular cortex

Abs: abstinence duration; ACC: anterior cingulate cortex; C: control group; DLPFC: dorsolateral prefrontal cortex; fMRI: functional magnetic resonance imaging; FOC: frontal operculum cortex; ICC: intracalcarine cortex; IFG; inferior frontal gyrus; IPS: intraparietal sulcus; ITG: inferior temporal gyrus; L: left; LOC: lateral occipital cortex; MA: methamphetamine group; MFG: middle frontal gyrus; MTG: middle temporal gyrus; PCC: posterior cingulate gyrus; PCG: precentral gyrus; PFC: prefrontal cortex; PPC: posterior parietal cortex; PPG: posterior parahippocampal gyrus; R: right; ROI: region of interest; RT: reaction time; SFG: superior frontal gyrus; SMA: supplementary motor area; STG: superior temporal gyrus; TOFC: temporal-occipital fusiform cortex; VLPFC: ventrolateral prefrontal cortex.

### 4.1. fMRI Studies of Decision-Making and Reward Processing in MA Abusers

#### 4.1.1. fMRI Studies of Decision-Making in MA Abusers

The earliest fMRI studies in MA dependence investigated decision-making amongst MA abusers [7,148,149]. Using a decision-making task during an fMRI session, Paulus and colleagues demonstrated deficits in decision-making and corresponding decreases in cortical activation in recently abstinent MA-dependent males [7]. The task consisted of two conditions, namely the two-choice prediction task and the two-choice response task, during which subjects made a choice between two possible outcomes, and 50% of choices were reinforced as if they were ‘correct’ predictions [7]. MA abusers were more significantly influenced than their comparison subjects by the immediately preceding outcome in their following choice; specifically, they were more likely to implement a “win-stay/lose-shift” strategy [7]. This dysfunction in decision-making processes was associated with shorter abstinence periods amongst MA abusers [7]. During the two-choice prediction task relative to the two-choice response task, both MA and control groups exhibited activations in the prefrontal and parietal cortices [7]. However, the magnitude of activation in the left middle frontal gyrus (Brodmann area (BA) 9), a part of the DLPFC, was greater in controls than MA abusers [7]. Additionally, controls showed ventromedial cortex activations including the right orbitofrontal gyrus (BA 10,11). In contrast, there was a lack of OFC activation amongst the MA abusers, which was associated with longer duration of MA use [7]. To sum up, during the process of decision-making recently abstinent MA abusers were less capable than controls of inhibiting the strategy of prediction based on preceding outcomes [7]. That is, MA abusers were more influenced by success relative to controls; a deficit which was related to dysfunctions of the DLPFC and OFC [7].

In a follow-up study [148], Paulus and colleagues assessed whether the extent of predictability differentially influenced decision-making of MA-dependent males, by varying the success rate on the two-choice prediction task described in their previous study [7]. MA abusers were again more likely to use the “win-stay/lose-shift” strategy in comparison with controls [148]. Significant group effects were found in fMRI activation; MA abusers exhibited less task-related activation in bilateral inferior PFC (medial frontal gyrus, BA10), DLPFC (middle and medial frontal gyri, BA 8,9), bilateral parietal cortex (BA 7/19), left post-central gyrus (BA 5) and left superior temporal gyrus (BA 6,9) [148]. The error-rate effect was significant in both MA and controls in the right insula (BA 13), right inferior frontal (BA 44,45), right middle frontal (BA 9) and left middle frontal gyri (BA 6,9). Lower error rates corresponded with higher task-related activation in both groups in all areas with the exception of the left middle frontal gyrus, and activation was lowest during the most unpredictable stimuli (50% error rate) [148]. However, a contrast of group by error-rate effect revealed that the increase in task-related activation at lower error rates was mostly carried by the control group and that MA abusers in fact had attenuated activation at lower error rates [148]. The group by error-rate contrast revealed higher activation in controls during low error rates in brain regions crucial for decision-making processes, namely the left medial frontal gyrus (BA 10), insula (BA 13), precuneus (BA 7) and inferior parietal lobule (BA 40) [148]. Conversely, MA abusers showed increased activation of these areas during the most unpredictable condition and were less engaged in processing success or failure relative to controls [148]. Subjects who had a longer duration of MA use showed decreased task-related activation in the left middle frontal gyrus (BA 9), ACC (BA 32) and left precuneus (BA 7) [148]. Moreover, longer abstinence periods were associated with higher activation in the left medial frontal gyrus (BA 8) [148]. The authors concluded that the increased neural activation in response to stimulus uncertainty in MA abusers reflected a “stimulus-driven” rather than an “outcome-driven” strategy during decision-making [148]. 

In a later study, Paulus and colleagues used the two-choice prediction task and the two-choice response task to investigate whether task-related activation on fMRI predicted relapse in a large group of MA-dependent males [149]. A group of 46 MA-dependent males were scanned at baseline and then followed up a year later to determine their abstinence status using a semi-structured questionnaire. Six subjects were lost to follow-up, 18 subjects relapsed and 22 subjects remained abstinent [149]. Similar to previously-reported results [7,155], the two-choice prediction task resulted in increased activation in the bilateral PFC, striatum, posterior parietal cortex (PPC) and anterior insula, relative to the two-choice response task [149]. MA abusers who relapsed exhibited lower task-related activation in the PFC, insula and parietal cortex in comparison with non-relapsing MA abusers [149]. A step-wise discriminant function analysis, with relapse status as the dependent measure and activation areas that differed between relapsing and non-relapsing MA as the independent measures, was used to evaluate which areas best predicted relapse. The analysis revealed that activation in the right insula, right posterior cingulate cortex (PCC) and right middle temporal gyrus best predicted relapsing (17 out of 18) and non-relapsing (20 out of 22) MA abusers [149]. Finally, a step-wise Cox regression analysis, with time to relapse as the dependent measure and activation areas that differed between relapsing and non-relapsing MA abusers as the independent measures, showed that the right middle frontal and temporal gyri and right PCC were the areas which best predicted time to relapse [149]. This study demonstrated that fMRI can be used for prediction of relapse in MA dependence; specifically, changes in fMRI activation during decision-making tasks may be a good predictor of relapse [149]. However, the use of a block-design in this study made it difficult to isolate a precise component of decision-making. The authors advised future studies to employ event-related paradigms to pinpoint aspects of decision-making most related to the prediction of relapse [149].

#### 4.1.2. fMRI Studies of Reward Processing in MA Abusers

Decision-making has been studied in relation to reward in MA dependence using the delay discounting task paired with fMRI [150,151]. When a choice between immediate and delayed rewards is presented, delayed rewards are not considered to be as valuable as immediate rewards of similar value [156,157]. The devaluation of future rewards is referred to as “delay discounting”. Generally, greater discounting has been associated with impulsive behaviours such as impulsive gambling or drug-taking with limited consideration of future rewards such as good health [158,159,160]. 

During a delay discounting task, the individual is required to make a series of choices between rewards immediately available and those of greater magnitude provided after some time delay [150]. Each individual’s discount rate is calculated using mathematical modeling of the rate at which they reduced the value of delayed rewards [156]. Monterosso and colleagues used this method and administered a delay discounting task to currently using MA-dependent and comparison subjects outside of the MRI scanner to generate a delay discounting probe task paired with fMRI [150]. Based on the subjects’ own discounting functions computed from the pre-scanning task, the fMRI task consisted of three conditions: “hard choice” requiring selection between immediate and delayed choices of similar reward magnitude, “easy choice” requiring selection between choices which dramatically differed in reward magnitude and a baseline “no choice” condition [150]. MA abusers exhibited more delay discounting than controls during the task conducted prior to scanning [150]. Across both groups, the “hard choice > no choice” contrast yielded activations in the ventrolateral PFC (VLPFC; BA 10,11,47), DLPFC (BA 9,46), dorsal ACC and areas surrounding the intraparietal sulcus (IPS; BA 40,7) [150]. Moreover, the “hard choice > easy choice” contrast resulted in activations in the right VLPFC and in a cluster which extended between the ACC and the supplementary motor area (SMA) [150]. Between-group comparisons revealed that controls had higher activations than MA abusers during the “hard choice > easy choice” contrast in the left DLPFC and right IPS [150]. Whereas neural recruitment was high during “hard choice” trials and minimal during the “easy choice” trials in controls, MA abusers experienced approximately equal levels of neural activation during both conditions, suggesting an inefficiency in frontoparietal circuitry [150]. A correlational analysis was conducted to assess the relationship between the degree of delay discounting and task-related activation. There were no significant correlations between the higher degree of delay discounting and inefficient frontoparietal processing during decision-making in currently abusing MA-dependent subjects [150]. The lack of difference in neural recruitment in MA abusers during “easy choice” *vs*. “hard choice” conditions may reflect procedural stimulus-directed rather than declarative outcome-directed behaviour [156], as has been previously suggested by Paulus and colleagues [148]. Findings from this study are limited by a small sample size and the inability to investigate fMRI response in the potentially task-relevant OFC, a region which is typically impacted by signal loss due to being located in proximity to air-filled sinuses which act as a susceptibility artifact [150]. 

Another study compared delay discounting and related cortical activation in fMRI between a group of abstinent MA abusers and comparison subjects [151]. Again, MA abusers exhibited greater delay discounting and chose more immediate rewards in comparison with controls [151]. “Easy choice” trials were eliminated from the fMRI analysis due to having much smaller contrast against control trials when compared to “hard choice *vs*. control” [151]. Across both groups, the “hard choice > control” contrast yielded higher activation in the bilateral dorsal ACC, medial superior frontal gyrus, PCC, V/DLPFC, right insula and PPC including the IPS [151]. When groups were compared, controls exhibited higher activation than MA abusers during the “hard choice > control” contrast in the bilateral precuneus, right caudate, right ACC and right DLPFC [151]. When subjects selected the delayed reward during “hard choice” trials, higher magnitudes of discounting were associated with increased activation in the right amygdala, superior frontal gyrus, PCC and PPC [151]. MA abusers had longer reaction times even during control trials and exhibited a smaller difference between delay discounting trials and control trials due to activating to a greater extent than controls during control trials. A similar finding has been previously reported by Monterosso and colleagues [150], and reflects an increased difficulty in decision-making processes by MA abusers relative to controls [151]. Increased activation of the amygdala, a component of the ventral affective system, was thought to occur in response to the selection of a delayed reward which is considered “an aversive choice” or a “loss” by heavy discounters such as MA abusers, who generally prefer immediate rewards [151]. The authors also inferred that in order to select the less common delayed rewards, steep discounters, mainly consisting of MA abusers in this sample, needed to activate frontoparietal circuits to exert control over amygdala activation [151].

The literature comparing activated neural circuits during decision-making in MA abusers and controls generally suggests reduced neural recruitment within the PFC, particularly in the DLPFC, of MA abusers [7,148,150,151,161].

### 4.2. fMRI Studies of Cognitive Control in MA Abusers

Inhibitory control is thought to be impaired in drug abusers, with a corresponding reduction in ACC activity [162]. Functional neuroimaging has been used to study inhibitory control in MA abusers using the go/nogo response inhibition task [152]. Leland and colleagues studied both inhibition- and predictive cue-related activation in two regions of interest (ROI) in the ACC; ventral and dorsal, in a group of MA abusers and their comparison subjects [152]. After being presented with an implicit predictive cue, MA abusers made less errors *i.e.*, had a lower false alarm rate, whereas controls’ false alarm rate was not different after predictive cueing [152]. An inhibition (nogo-go) contrast was constructed and yielded no between-group differences in activation, however in separate analyses; both groups exhibited increased activation in non-overlapping areas in the ventral ACC [152]. Of greater interest to the authors was the effect of predictive cueing on activation in the ACC, hence a cueing (cue-noncue) contrast was computed [152]. A between-group analysis of the cueing contrast resulted in increased activation in clusters in both the ventral and dorsal ACC. However, separate group analyses revealed no cueing-related change in activation in controls; the effect was carried by the higher cueing-related activation in MA abusers within both the ventral and dorsal ACC [152]. A regression analysis of cueing-related activation in the ventral and dorsal ACC of MA abusers and a difference score of cued *vs*. uncued false alarm rates revealed that higher cueing-related activation in the ventral ACC of MA abusers was associated with the largest cue-related decrease in false alarm rate [152]. Despite the limited behavioural differences in task performance between MA abusers and controls, MA abusers exhibited higher inhibition-related activation of the dorsal and ventral ACC [152]. Moreover, MA abusers showed a larger benefit from predictive cueing on false alarm rate which was associated with increased ACC activity, thus conveying attenuated inhibitory difficulties in the presence of advance warning in the form of an implicit predictive cue [152]. 

The Stroop task has been used in conjunction with fMRI to study cognitive control in MA abusers [153,154]. Salo and colleagues used an fMRI-coupled modified Stroop colour-word task to study differences in selective attention performance and related brain activations in a group of abstinent MA abusers and their comparison subjects [153]. In previous studies, this version of the Stroop task has produced activation of the PFC and ACC [163,164], hence this study limited its inference to ROIs which corresponded to these regions [153]. The paradigm consisted of congruent (nonconflict) trials, where the word and font colour matched and incongruent (conflict) trials, where the word and font colour did not match [153]. The first effect of interest was the conflict effect, which was measured by the contrast “incongruent-congruent” [153]. The second effect of interest was the trial-to-trial adjustment effect, measured by comparing sequences of incongruent trials preceded by incongruent trials with those of incongruent trials preceded by congruent trials, *i.e.*, “incongruent/incongruent-congruent/incongruent” [153]. There were significant activations in the ACC during the conflict effect contrast in all subjects with no significant differences between groups, corresponding with the higher conflict resolution demands during incongruent trials [153]. Behavioural results showed no between-group difference in Stroop conflict effect or accuracy rates [153]. However, there was a reduction in the reaction time adjustment effect with a corresponding decrease in activation of the right PFC, particularly in BA 6, in MA abusers relative to controls [153]. Controls were able to utilise previous exposure to a conflict event to improve their conflict processing resulting in faster reaction times on subsequent trials coupled with increased activation in the right PFC [153]. In contrast, MA abusers did not exhibit this advantage and displayed a trend for slower reaction times to incongruent trials preceded by incongruent trials, simultaneously exhibiting little or no activation in the PFC [153]. Controls activated the PFC during conflict processing, thereby engaging top-down control [163], whilst MA abusers exhibited deficits in trial-to-trial adjustments which reflected sub-optimal behavioural modification with corresponding deficits in the PFC [153]. Deficits in the frontal cortex related to behavioural modification in MA abusers were thought to be related to their inability to maintain goal-directed behaviour, reflecting poor cognitive control which potentially contributes to drug-seeking behaviour and relapse [153]. MA-dependent participants recruited in this study had been abstinent for anywhere between two and twelve months (mean ± SD, 4.1 ± 2.8 months) [153]. However, the first weeks of abstinence are thought to be important for initial engagement and subsequent retention in treatment programs [154]. Hence, the relationship between cognitive control deficits and relapse may be better assessed during early abstinence from the drug of abuse [154]. 

Nestor and colleagues studied cognitive control in MA abusers in their first week of abstinence (4–7 days) using a blocked Stroop colour-word interference task [154]. The task consisted of two conditions, congruent and incongruent colour words [154]. First, a whole brain analysis was carried out, which did not take individual reaction times into account [154]. However, due to differences in behavioural performance between groups, a second analysis was carried out controlling for reaction time by including mean reaction time during each condition as a covariate of no interest [154]. In doing so, this analysis took into consideration the potential confounding effect of differing performance levels [154], or time-on-task, an effect which has been previously reported in fMRI studies of cognitive control [165]. For each analysis, three contrasts of interest were constructed for both conditions and the Stroop effect (incongruent–congruent) [154]. The first analysis, not taking into account the effect of reaction times, yielded significant group differences in activations only on the Stroop effect contrast [154]. In comparison with controls, MA abusers exhibited reduced activation in the left cerebellum and greater activation in several brain regions including the PCC, bilateral ACC and left medial frontal cortex [154]. Higher activity in the PCC of MA abusers during the Stroop effect may correspond to error processing. The PCC has been shown to be active during sensory arousal in cocaine abusers [136], following response errors in healthy volunteers [166] and, as mentioned in the previous section of this review, it was reported to be a region of abnormally high glucose metabolism during error processing [104]. Increased activation in the ACC of MA abusers during the Stroop effect may be related to the behavioural results which reflect compromised conflict monitoring in MA abusers [167]. Moreover, greater activity within the medial frontal cortex may be related to monitoring task performance outcomes, including detection of performance errors, response conflict and unfavourable outcomes [168,169]. 

The second analysis, examining group differences whilst controlling for reaction times, yielded significant group differences on the incongruent condition contrast only [154]. Relative to controls, MA abusers did not exhibit higher activation in any brain region during either condition [154]. However, they exhibited lower activation in the right cortex, for example in the inferior frontal gyrus, SMA/ACC, and anterior insular cortex [154]. The inferior frontal gyrus plays a central role in response inhibition [170,171] as well as interference suppression [171], therefore reduced activity of the inferior frontal gyrus may be explained by the behavioural results of poor cognitive control in MA abusers reported in this study [154]. Hypoactivation of the SMA of MA abusers during the incongruent condition was thought to be related to impaired planning of motor actions [172], as well as response to conflict [173] and action-based decision-making [174]. Whereas hypoactivation of the ACC may be linked to task performance during conditions of high conflict [175,176], such as the incongruent condition of the Stroop task, where there is increased likelihood of making errors [177]. The insula has been implicated in error-related processes [178,179], and plays a central role in reward-related decision-making processes by integrating bodily (interoceptive) states into conscious feelings such as urges to take drugs [180]. The reduction in insular activation of MA abusers reported in this study [154] may be related to response inhibition-related errors [178]. Although this study was limited by a small sample size, the blocked paradigm design in conjunction with fMRI results in greater power of signal detection in comparison with event-related designs, potentially allowing for a smaller sample size [181]. Nonetheless, the use of a blocked paradigm precluded analyses of “conflict adaptation effects” [182] and trial-to-trial adjustment to previous exposure of conflict as reported by Salo and colleagues [153,163]. Finally, given the short duration of abstinence from MA (4–7 days), withdrawal effects may have impacted the results of this study. However, the authors have previously shown a lack of association between abstinence duration and cognitive performance during the first week of abstinence from MA [183]. 

In summary, fMRI has been a useful tool for the study of neural activation of MA abusers during cognitive tasks. Using fMRI, MA abusers have been shown to exhibit cortical hypoactivation corresponding with deficits in decision-making, being more likely to employ a stimulus-driven rather than outcome-driven strategy [7,148]. Moreover, fMRI has been shown to be a potentially useful tool in predicting relapse in MA dependence [149]. Processing of delayed rewards has been associated with deficits in fronto-parietal circuitry and subcortical structures including the caudate and amygdala amongst MA abusers [150,151]. Lastly, the literature on cognitive control in MA abusers studied with fMRI within this review suggests deficits in cognitive control and corresponding dysfunctions in activation of frontal brain regions integral to cognitive control during response inhibition and conflict adaptation as measured by the go/nogo and colour-word Stroop tasks [152,153,154]. 

## 5. Structural Brain Abnormalities in MA Abusers Using Structural MRI

There have been few studies investigating structural changes in the brains of MA-dependent individuals with variable abstinence periods. Although all structural MRI studies which measured total brain volumes reported no differences between MA-dependent individuals compared with controls [184,185,186,187], regional structural differences in both grey matter (GM) and white matter (WM) have been found in most of these studies in MA abusers, most of whom had been voluntarily abstinent or detoxified for the purposes of these studies. In this section of the review, GM structural changes will be discussed, followed by structural changes in the WM of MA abusers in comparison with healthy controls. Study specifications and main findings are summarised in Table 3.

**Table 3:** Summary of study specifications and findings from structural MRI studies of MA dependence.

Authors	Groups	Age (year) (Mean ± SD)	Male	MA Use Variables (Mean ± SD)	Tesla (T)	**Assessed? or Regions**	Main Findings
**Thompson *et al.* 2004 [186]**	22 MA	35.3 ± 1.66	15	MA use (10.5 ± 1.1 years)	3	Cortical GM mapping of cortex, hippocampus, WM and ventricles	No group difference in total or cerebral GM
							↓ GM in MA relative to C in R ACC and PCC, R subgenual cortex, R paralimbic belts
				MA dose (g/week): 3.44 ± 0.79			
	21 C	31.9 ± 1.47	10				↑ WM in temporal and occipital regions of MA relative to C
						GM and WM	
				CumMA (g): 1878 ± 126			↓ hippocampal vol in MA users relative to C
**Chang *et al.* 2005 [184]**	50 MA	32.1 ± 7.1	24	MA use >2 years (110 ± 68 months)	1.5	ROIs in the caudate, putamen, globus pallidus, thalamus, midbrain, cerebellar vermis and corpus callosum	No group difference in whole brain volumes
							↑ Putamen vol (L +10.3%; R +9.6%) of MA relative C
							↑ GP vol (L +9.3%; R +6.6%) of MA relative to C
						Neuropsychological test battery	Putamen and GP volumes negatively correlated with CumMA (g) and positively correlated with verbal fluency and speeded motor tasks
				Abs > 1 week (4 ± 6.2 months)			
	50 C	31.7 ± 7.4	24			GM and WM	
				CumMA (g): 4519 ± 5730		Abs effects	
						Gender effects	
							↑ midposterior corpus callosum vol (+9.7%) of F MA
**Oh *et al.* 2005 [188]**	27 MA	36.7 ± 5.6	23	MA use: 21 ±35 months	3	Automated shape analysis of the corpus callosum (callosal width)	No group difference in corpus callosum volumes
				Abs: 20.5 ± 35.4 months			↑ Genu curvature in MA relative to C
						WM only	↓ width of posterior midbody and isthmus of corpus callosum of MA relative to C
	18 C	33.6 ± 6.7	14				
				MA dose (g/day): 0.63 ± 0.50			
				CumMA (g): 334 ± 506			
**Jernigan *et al.* 2005 [189]**	21 MA	38.2 ± 7.7	17	MA use >6 years (12.1 ± 4.1 years)	1.5	Semi-automatic tissue segmentation and ROIs in cortex, caudate, putamen, globus pallidus, nucleus accumbens, thalamus, amygdala and hippocampus	↑ GM vol in parietal lobe of MA relative to C, correlated with cognitive impairment
	22 MA + HIV	39.0 ± 6.7	21				
	30 HIV	38.1 ± 6.0	28	Abs > 10 days (94 ± 89 days)			
						GM only	↑ GM vol in caudate, putamen, globus pallidus and nucleus accumbens
	30 C	38.1 ± 10.5	17	CumMA (g): 4930 ± 94			
						Gender effects	
**Bae *et al.* 2006 [190]**	33 MA	32.8 ± 6.4	22	MA use: 59.3 ± 36.0 months	3	WM hyperintensity prevalence, severity located graded on T2-weighted images	↑ WM hyperintensities prevalence in MA (33%) relative to C (3%)
				Abs: 18.4 ± 28.7 months			
						Deep (all lobes + insula) and periventricular (frontal and non-frontal)	
				MA does (g/day): 0.62 ± 0.46			
							↑ severity of all, deep and periventricular WM hyperintensities in MA relative to C, m MA > F MA, positively correlated with CumMA (g)
	32 C	32.4 ± 6.2	21				
				CumMA (g): 292.1 ± 246.8			
						Abs effects	
						Gender effects	
**Kim *et al.* 2006 [191]**	11 short-Abs MA (<6 months)	37.9 ± 6.0	11	Mean MA use: 64 ± 44 months	3	Whole brain VBM	↓ GMD (−10.3%) in R MFG, BA10 (short-Abs MA < long-Abs < control)
				Abs: All MA: 20 ± 33.5 months			
						GM only	
	18 long-Abs MA (>6 months)						↑ total errors on the WCST (short-Abs > long-Abs > control)
						Abs effects	
				Short-Abs MA: 2.6 ± 1.6 months			
						WCST	
						Trailmaking test	
		35.6 ± 5.2	16				
				Long-Abs MA: 30.6 ± 39.2 months		Stroop test	
		33.2 ± 6.5	15				↓ GM in R MFG negatively correlated with total errors on the WCST
	20 C			CumMA (g): 276 ± 236			
**Chung *et al.* 2007 [192]**	32 MA	34.0 ± 7.5	23	MA use (M: 75 ± 51 months, F: 47 ± 50 months)	3	FA using DTI in frontal WM ROIs	↓ FA (WM integrity) in frontal WM of MA relative to C (M > F)
						WM only	
				Abs > 1 month (M: 24 ± 38 months, F: 43 ± 66 months)			
						WCST	↑ total errors on the WCST of MA relative to C (M > F) negatively correlated with FA
	30 C	31.6 ± 6.7	20			Gender effects	
				CumMA (g): M: 412 ± 543, F: 133 ± 150			
**Schwartz *et al.* 2010 [187]**	61 MA	33.4 ± 8.4	31	Age at first MA use: 19.1 ± 7.6 years	3	Whole brain VBM	No group difference in whole brain volumes
						Whole brain volumetric analysis	
	44 C	34.1 ± 10.7	22			GM only	↓ GMD in R insula (−9.7%), L insula (−9.6%), L MFG (−12.6%) in MA relative to C
				Abs: 63.7 ± 32.7 days		Abs effects	
						Gender effects	↑ GMD in L inferior semilunar lobule, part of the cerebellum (+10%) in MA
						Impulsivity (DDT)	↑ impulsivity in MA (correlations presented in-text)
**Nakama *et al.* 2011 [193]**	34 MA	33.1 ± 8.9	21	MA use: 120 months (60–180 months)	3	Regional cortical GM within ROIs in 6 main lobes (frontal, temporal, parietal, occipital, limbic and insular) and 17 sub-regions	↓ GM vol in MA in all 6 main lobes relative to C, significance not reached in frontal and occipital lobes
				Abs: 18 days (range 0–120 days			
						GM only	↓ GM vol in MA in 17 sub-regions relative to C, significance reached in DLPFC, OFC, PFC and superior temporal subregions
	31 C	35.7 ± 8.4	23				
				CumMA (g): 787 (364–2737)		Age effects	
							GM vol negatively correlated with age in frontal, occipital, temporal and insular lobes in MA

Abs: abstinence/abstinent; ACC: anterior cingulate cortex; C: control group; CumMA (g): lifetime cumulative methamphetamine dose in grams; DLPFC: dorsolateral prefrontal cortex; DDT: delay discounting task; DTI: diffusion tensor imaging; F: female; FA: fractional anisotropy; GM: grey matter; GMD: grey matter density; GP: globus pallidus; HIV: human immunodeficiency virus; L: left; M: male; MA: methamphetamine group; MFG: middle frontal gyrus; OFC: orbitofrontal cortex; PCC: posterior cingulate gyrus; PFC: prefrontal cortex; R: right; ROI: region of interest; SFG: superior frontal gyrus; VBM: voxel-based morphometry; vol: volume(s); WCST: Wisconsin card sorting test; WM: white matter.

### 5.1. Structural MRI Studies of GM Changes in MA Abusers

#### 5.1.1. Structural MRI Studies of Cortical GM Changes in MA Abusers

Most studies reporting GM changes in MA abusers found a reduction in cortical GM [186,187,191,193], with the exception of one which reported an increase in parietal GM of abstinent MA abusers [189]. Higher parietal GM volume was associated with higher levels of neurocognitive impairment, possibly reflecting a neuroadapative compensatory response to MA-induced toxicity [189]. The increase in parietal lobe volume may have been a consequence of MA-related microgliosis, astrocytosis and glutamatergic excitotoxicity which have been observed in parietal regions in animal studies [88,194]. The authors discussed the possibility that neurodegenerative changes may have been present in their sample of MA abusers, but were obscured by mechanisms which lead to the observed volume increase in the parietal lobes [189]. 

To date, the only published study examining structural GM changes in active (non-abstinent) MA abusers was that of Thompson and colleagues’, who compared the brains of actively-using chronic MA abusers with age-matched controls [186]. Thompson and colleagues used GM mapping, a high resolution surface-based computational image analysis of structural MRI technique to investigate structural GM changes in the brains of MA abusers who used MA on most of the 30 days prior to entering the study [186]. They found no group differences in total cerebral volumes or total GM [186]. However, they noted severe GM deficits in the right hemisphere of chronic MA abusers (11.3% below control levels), mainly in the cingulate, limbic and paralimbic cortices, as well as hippocampal volumes (7.8% below control levels) [186]. Interestingly, GM deficits in the right ACC and PCC overlapped regions of abnormal glucose metabolism during a vigilance task in a previously published study which recruited some of the same subjects [103]. This study did not examine striatal volumes and hence inference in these areas was limited. Additionally, smoking may have confounded the results as it was more prevalent among the MA-dependent group than the control group [186]. Smoking has been shown to be associated with lower GM density (GMD) in the PFC, dorsal ACC and the right cerebellum [195], and its effects were not entirely separated from those of MA in the statistical analysis [186].

Another study which found GM deficits in the MA abusers was that of Schwartz and colleagues who found reduced GMD in the bilateral insula and left middle frontal gyrus of abstinent MA abusers in comparison with healthy controls [187]. Thinning of the insular cortex was thought to underlie conscious drug seeking and the behavioural biases associated with drug addiction [180,187], whereas GM reduction in the middle frontal gyrus was thought to be associated with cognitive control deficits in MA abusers [187]. This study also reported increased impulsivity amongst the MA group, as measured by the delay discounting task, whereby MA abusers were more likely to choose smaller immediate rather than larger delayed rewards [187]. Across both groups, impulsivity was associated with increased GM in the PCC and bilateral putamen extending into the left ventral striatum, and decreased GM in the left superior frontal gyrus near the frontal pole [187]. Nevertheless, causality remained unclear; the authors could not determine whether these results were caused or preceded by differences in impulsivity between addicts and non-addicts [187]. A within-group analysis was carried out in MA abusers only to investigate the relationship between GMD and duration of abstinence [187]. Most notably, longer abstinence durations were associated with increased GMD in the bilateral amygdala and decreased GMD in the right middle frontal gyrus [187]. Smaller amygdala volumes have been associated with increased craving and predictive of relapse [196], whilst the middle frontal gyrus is part of the DLPFC, necessary for controlling impulsive behaviours such as relapse into drug use [197,198]. 

Kim and colleagues investigated the effect of duration of abstinence from MA on GM volumes using voxel-based morphometry (VBM), as well as frontal executive function using the WCST and other cognitive tests [191]. They compared MA abusers who were short-term abstinent (mean abstinence 2.6 ± 1.6 months) with those who were long-term abstinent (mean abstinence 30.6 ± 39.2 months) and a group of healthy controls [191]. There were significant group differences both in GMD in the right middle frontal gyrus (MA < controls by 10.3%) and total errors on the WCST between the three groups, the latter reflecting impairment in frontal executive function [191]. Although long-term abstinent abusers were impaired in comparison with control subjects, they had significantly less GMD decrease and less total errors on the WCST compared to short-term abstinent abusers [191]. The authors suggested that both effects partially recover after abstinence from MA for six months or longer [191] in a similar fashion to the recovery, after 12–17 months of abstinence, of striatal DAT levels [99] and thalamic glucose metabolism [102]. Due to the cross-sectional design of this study, the authors were unable to decipher whether decreases in frontal GMD were caused by MA use, and whether short-term abstinence was associated with the lowest GMD or if decreased frontal GMD preceded becoming MA-dependent, and increased likelihood of relapse [191]. In saying that, the researchers ensured no significant differences existed in the MA use variables, thus supporting the explanation of GM recovery with long-term abstinence over differences in the preceding constitution [191]. 

Using ROI-based methods to determine regional differences in volume of cortical GM between abstinent MA abusers and controls as well as their interaction with age, Nakama and colleagues found smaller cortical GM volumes in six main lobes and 17 sub-regions of the cortical GM [193]. MA abusers were found to have smaller frontal, temporal, parietal, limbic, insular and occipital lobes, as well as reduced GM in the DLPFC, OFC, PFC and superior temporal regions [193]. This was the first study to assess the effect of MA dependence on the aging brain, and it reported a pattern of more rapid GM loss in frontal, temporal, occipital and insular lobes amongst MA abusers resembling that of accelerated aging [193]. It is possible that this age-related GM loss in MA abusers may lead to a reduction in brain reserve capacity which in turn may increase their vulnerability to deficits in cognitive function [199]. This study was cross-sectional and therefore could not provide direct links between age and brain volume reductions in MA abusers [193]. Moreover, it did not evaluate the effect of MA use on subcortical volumes, and the moderate sample size did not allow for sub-group analyses of abstinence and gender effects [193]. 

Most structural studies investigating the cerebral cortex in MA abusers reported GM deficits in frontal cortical areas [186,187,191,193]. Findings of deficits in frontal GM are in line with non-volumetric neuroimaging studies mentioned earlier in this review. For example, an fMRI study by Paulus and colleagues reported lower activation in the DLPFC of MA abusers during a decision-making task in comparison with non-drug abusers [7]. Using PET, MA abusers were reported to have lower DAT levels [101] as well as altered glucose metabolism [103] in frontal brain regions. Moreover, abstinent MA abusers were found to have a metabolite abnormality in frontal brain regions, as measured by magnetic resonance spectroscopy, revealing decreased levels of the *N*-acetylaspartate metabolite, a marker of neuronal viability [126]. 

#### 5.1.2. Structural MRI Studies of Subcortical GM Changes in MA Abusers

Striatal volumes have also been studied in MA abusers, using a combination of interactive semi-automatic tissue segmentation and ROI analysis methods [184,189]. Abstinent MA abusers were found to have larger bilateral volumes of the caudate, nucleus accumbens [189], putamen and globus pallidus [184,189] in comparison with healthy controls [184,189]. Chang and colleagues [184] also assessed gender differences in brain volumes and observed larger whole brain and regional volumes in men compared with women in the cerebellum, thalami and caudate nuclei; however, there were no significant gender effects in the putamen or globus pallidus. GM volume increases in the striatum of MA abusers were thought to result from inflammatory processes and reactive gliosis [87,88,129]. In MA abusers, larger putamen volumes were associated with lower lifetime cumulative MA use and improved performance on verbal fluency and speeded motor tasks [184]. Hence, it was thought that enlargement of the striatum may reflect a compensatory response to chronic MA-induced injury during earlier phases of drug dependence, an adaptation in order to maintain cognitive function [184,189]. However, this adaptation may fail to maintain both function and structural integrity of the striatum following prolonged use [184]. 

### 5.2. Structural MRI Studies of WM Changes in MA Abusers

WM differences in MA abusers were reported in several studies, including WM hypertrophy (7% above control levels) in temporal and occipital regions which were adjacent to some of the same regions of cortical and hippocampal GM loss in the same sample [186]. Therefore, WM loss may be partly explained by the GM loss in surrounding areas [186]. However, additional processes were thought to be involved including MA-induced gliosis leading to adaptive glial changes, altered myelination and developmental differences [186,200]. Female MA abusers have been shown to have 9.7% larger volumes of the midposterior corpus callosum in comparison with age- and gender-matched controls [184]. It was thought that this increase in WM volume in MA-using females may be related to the previously reported increase in glucose metabolism and perfusion in the parietal lobes of abstinent MA abusers [95,108,201], especially since the parietal fibers cross in the midposterior portion of the corpus callosum and this effect was found to be more prominent in the female abusers [201]. In addition to volume changes, abstinent MA-dependent males were found to have a higher prevalence and greater severity of WM hyperintensities, primarily in the frontal lobes, in comparison with female MA abusers and healthy controls [190]. WM hyperintensities in MA abusers are thought to be related to MA-induced deficits in cerebral perfusion [190]. Females are thought to be less affected by these structural abnormalities due to oestrogen’s protective effects against ischaemic damage [202,203], including those associated with MA [133,204]. Chung and colleagues reported decreased frontal WM integrity (as measured by fractional anisotropy using diffusion tensor imaging) in MA abusers who were abstinent for a month or longer [192]. However, this abnormality was only significant in male abusers, once again suggesting a protective factor in female abusers [192]. Despite reporting no group differences in WM volume between MA abusers and controls, corpus callosum shape differences were reported in a sample of MA abusers consisting mainly of males (89%) [188]. In comparison with controls, MA abusers showed increased curvature of the genu and decreased width of the posterior midbody and isthmus of the corpus callosum [188]. The WM abnormalities located in the frontal and parietal cortices of MA abusers may underlie functional abnormalities observed in these areas [188]. 

In summary, structural MRI studies in MA dependence have reported a variety of structural deficits. GM deficits were found in the frontal, insular, cingulate, temporal and occipital cortices [186,187,191,193] and GM gains were found in the parietal lobe [189] and striatum [184,189]. MA abusers were reported to have WM hypertrophy in temporal and occipital regions [186], increased prevalence and greater severity of WM hyperintensities [190], abnormalities in corpus callosum shape [188], and female abusers had larger volumes of the midposterior corpus callosum [184]. Despite this finding, male MA abusers were found to have more WM deficits than female abusers, with higher prevalence and severity of WM hyperintensities [190] and decreased WM integrity [192] in the frontal lobes. Female abusers were thought to have protective effects against structural abnormalities possibly owing to their higher oestrogen levels [133,204]. The variability of structural differences reported by different studies may be due to differences in sample size, data analysis methods, MA use variables and active use *versus* abstinence, including duration of abstinence.

## 6. Conclusions

In summary, MA is a powerful psychostimulant whose abuse has escalated globally over recent years and its abuse is known to cause disruptions in monoaminergic transmission, particularly degeneration of DA and 5-HT terminals, and cell death in rodent models of MA neurotoxicity. The brains of MA-dependent individuals have been studied using advanced neuroimaging techniques. This article reviewed evidence from PET, fMRI and structural MRI studies in MA addiction. 

PET studies in MA dependence reported deficits in presynaptic and postsynaptic dopaminergic and serotonergic neurotransmission, whereby reductions in the levels of DA, DAT, VMAT-2, D2 receptors and 5-HTT were observed, mainly in the striatum of MA abusers. In contrast, D3 receptor levels are thought to be increased in MA dependence. Moreover, using PET, glucose metabolism was found to be lower in the striatum, thalamus, insula and ACC but higher in the parietal cortex and ventral striatum of MA abusers. Deficits in thalamic glucose metabolism were thought to be reversible, whilst those in the striatum were thought to persist for prolonged periods following abstinence. 

Behavioural deficits have been observed in MA abusers during tasks of decision-making, reward processing and cognitive control with corresponding dysfunctions in cortical and subcortical activations as measured by fMRI. 

Structural GM deficits were found in cortical areas of MA abusers, whilst GM volume gains were observed in striatal structures, possibly reflecting a compensatory response to maintain cognitive function. Abnormalities in WM structure in MA abusers included WM hypertrophy, higher prevalence and greater severity of WM hyperintensities and abnormalities in corpus callosum shape. 

Individually, these studies highlighted common brain regions that are affected by MA, with the most common being frontal cortical and striatal structures, however many discrepancies were noted. There is a shortage of longitudinal studies of MA addiction which follow at-risk individuals into drug use or those which follow active users to abstinence, and there is a need for more research which combines multiple neuroimaging techniques in order to further understand the connections between observed changes.

## Abbreviations

5-HT: serotonin
5-HTT: serotonin transporter
ACC: anterior cingulate cortex
DA: dopamine
DAT: dopamine transporter
DLPFC: dorsolateral prefrontal cortex
DTBZ: dihydrotetrabenazine
FDG: fluorodeoxyglucose
fMRI: functional magnetic resonance imaging
GM: grey matter
GMD: grey matter density
IPS: intraparietal sulcus
MA: methamphetamine
MAO: monoamine oxidase
McN-5652: *trans*-1,2,3,5,6,10-β-hexahydro-6-[4-(methylthio)phenyl]pyrrolo-[2,1-*a*]isoquinoline
MRI: magnetic resonance imaging
NA: noradrenaline
NAT: noradrenaline transporter
NO: nitric oxide
OFC: orbitofrontal cortex
PCC: posterior cingulate cortex
PET: positron emission tomography
PFC: prefrontal cortex
PHNO: propyl-hexahydro-naphtho-oxazin
PPC: posterior parietal cortex
ROI: region of interest
SMA: supplementary motor area
TH: tyrosine hydroxylase
VBM: voxel-based morphometry
VLPFC: ventrolateral prefrontal cortex
VMAT-2: vesicular monoamine transporter-2
WM: white matter

## References

[1] United Nations Office on Drugs and Crime (2007). World Drug Report 2007.

[2] United Nations Office on Drugs and Crime (2010). World Drug Report 2010.

[3] Busto U., Bendayan R., Sellers E.M. (1989). Clinical pharmacokinetics of non-opiate abused drugs. Clin. Pharmacokinet..

[4] Barr A.M., Panenka W.J., MacEwan G.W., Thornton A.E., Lang D.J., Honer W.G., Lecomte T. (2006). The need for speed: An update on methamphetamine addiction. J. Psychiatry Neurosci..

[5] Cruickshank C.C., Dyer K.R. (2009). A review of the clinical pharmacology of methamphetamine. Addiction.

[6] Gonzalez R., Rippeth J.D., Carey C.L., Heaton R.K., Moore D.J., Schweinsburg B.C., Cherner M., Grant I. (2004). Neurocognitive performance of methamphetamine users discordant for history of marijuana exposure. Drug Alcohol Depend..

[7] Paulus M.P., Hozack N.E., Zauscher B.E., Frank L., Brown G.G., Braff D.L., Schuckit M.A. (2002). Behavioral and functional neuroimaging evidence for prefrontal dysfunction in methamphetamine-dependent subjects. Neuropsychopharmacology.

[8] Woods S.P., Rippeth J.D., Conover E., Gongvatana A., Gonzalez R., Carey C.L.,  Cherner M., Heaton R.K., Grant I., HIV Neurobehavioral Research Center Group (2005). Deficient strategic control of verbal encoding and retrieval in individuals with methamphetamine dependence. Neuropsychology.

[9] Barr A.M., Markou A., Phillips A.G. (2002). A “crash” course on psychostimulant withdrawal as a model of depression. Trends Pharmacol. Sci..

[10] Kuczenski R., Segal D.S., Cho A.K., Melega W. (1995). Hippocampus norepinephrine, caudate dopamine and serotonin, and behavioral responses to the stereoisomers of amphetamine and methamphetamine. J. Neurosci..

[11] Sulzer D., Sonders M.S., Poulsen N.W., Galli A. (2005). Mechanisms of neurotransmitter release by amphetamines: A review. Prog Neurobiol..

[12] Rothman R.B., Baumann M.H. (2003). Monoamine transporters and psychostimulant drugs. Eur. J. Pharmacol..

[13] Parker E.M., Cubeddu L.X. (1988). Comparative effects of amphetamine, phenylethylamine and related drugs on dopamine efflux, dopamine uptake and mazindol binding. J. Pharmacol. Exp. Ther..

[14] Schmitz Y., Lee C.J., Schmauss C., Gonon F., Sulzer D. (2001). Amphetamine distorts stimulation-dependent dopamine overflow: Effects on D2 autoreceptors, transporters, and synaptic vesicle stores. J. Neurosci..

[15] Saunders C., Ferrer J.V., Shi L., Chen J., Merrill G., Lamb M.E., Leeb-Lundberg L.M., Carvelli L., Javitch J.A., Galli A. (2000). Amphetamine-induced loss of human dopamine transporter activity: An internalization-dependent and cocaine-sensitive mechanism. Proc. Natl. Acad. Sci. USA.

[16] Iversen L. (2006). Neurotransmitter transporters and their impact on the development of psychopharmacology. Br. J. Pharmacol..

[17] Brown J.M., Hanson G.R., Fleckenstein A.E. (2001). Regulation of the vesicular monoamine transporter-2: A novel mechanism for cocaine and other psychostimulants. J. Pharmacol. Exp. Ther..

[18] Rothman R.B., Partilla J.S., Baumann M.H., Dersch C.M., Carroll F.I., Rice K.C. (2000). Neurochemical neutralization of methamphetamine with high-affinity nonselective inhibitors of biogenic amine transporters: A pharmacological strategy for treating stimulant abuse. Synapse.

[19] Khoshbouei H., Wang H., Lechleiter J.D., Javitch J.A., Galli A. (2003). Amphetamine-induced dopamine efflux. A voltage-sensitive and intracellular Na^+^-dependent mechanism. J. Biol. Chem..

[20] Mantle T.J., Tipton K.F., Garrett N.J. (1976). Inhibition of monoamine oxidase by amphetamine and related compounds. Biochem. Pharmacol..

[21] Mandell A.J., Morgan M. (1970). Amphetamine induced increase in tyrosine hydroxylase activity. Nature.

[22] Rothman R.B., Baumann M.H., Dersch C.M., Romero D.V., Rice K.C., Carroll F.I., Partilla J.S. (2001). Amphetamine-type central nervous system stimulants release norepinephrine more potently than they release dopamine and serotonin. Synapse.

[23] Makisumi T., Yoshida K., Watanabe T., Tan N., Murakami N., Morimoto A. (1998). Sympatho-adrenal involvement in methamphetamine-induced hyperthermia through skeletal muscle hypermetabolism. Eur. J. Pharmacol..

[24] Wise R.A. (1996). Neurobiology of addiction. Curr. Opin. Neurobiol..

[25] Kogan F.J., Nichols W.K., Gibb J.W. (1976). Influence of methamphetamine on nigral and striatal tyrosine hydroxylase activity and on striatal dopamine levels. Eur. J. Pharmacol..

[26] Seiden L.S., Fischman M.W., Schuster C.R. (1976). Long-term methamphetamine induced changes in brain catecholamines in tolerant rhesus monkeys. Drug Alcohol Depend..

[27] Wagner G.C., Seiden L.S., Schuster C.R. (1979). Methamphetamine-induced changes in brain catecholamines in rats and guinea pigs. Drug Alcohol Depend..

[28] Finnegan K.T., Ricaurte G., Seiden L.S., Schuster C.R. (1982). Altered sensitivity to *d*-methylamphetamine, apomorphine, and haloperidol in rhesus monkeys depleted of caudate dopamine by repeated administration of *d*-methylamphetamine. Psychopharmacology.

[29] Preston K.L., Wagner G.C., Schuster C.R., Seiden L.S. (1985). Long-term effects of repeated methylamphetamine administration on monoamine neurons in the rhesus monkey brain. Brain Res..

[30] Preston K.L., Schuster C.R., Seiden L.S. (1985). Methamphetamine, physostigmine, atropine and mecamylamine: Effects on force lever performance. Pharmacol. Biochem. Behav..

[31] McCann U.D., Ricaurte G.A. (2004). Amphetamine neurotoxicity: Accomplishments and remaining challenges. Neurosci. Biobehav. Rev..

[32] Caldwell J. (1976). The metabolism of amphetamines in mammals. Drug Metab. Rev..

[33] Cappon G.D., Pu C., Vorhees C.V. (2000). Time-course of methamphetamine-induced neurotoxicity in rat caudate-putamen after single-dose treatment. Brain Res..

[34] Guilarte T.R., Nihei M.K., McGlothan J.L., Howard A.S. (2003). Methamphetamine-induced deficits of brain monoaminergic neuronal markers: Distal axotomy or neuronal plasticity. Neuroscience.

[35] Hotchkiss A.J., Gibb J.W. (1980). Long-term effects of multiple doses of methamphetamine on tryptophan hydroxylase and tyrosine hydroxylase activity in rat brain. J. Pharmacol. Exp. Ther..

[36] Ricaurte G.A., Schuster C.R., Seiden L.S. (1980). Long-term effects of repeated methylamphetamine administration on dopamine and serotonin neurons in the rat brain: A regional study. Brain Res..

[37] Richards J.B., Baggott M.J., Sabol K.E., Seiden L.S. (1993). A high-dose methamphetamine regimen results in long-lasting deficits on performance of a reaction-time task. Brain Res..

[38] Melega W.P., Lacan G., Desalles A.A., Phelps M.E. (2000). Long-term methamphetamine-induced decreases of [(11)C]WIN 35,428 binding in striatum are reduced by GDNF: PET studies in the vervet monkey. Synapse.

[39] Melega W.P., Jorgensen M.J., Lacan G., Way B.M., Pham J., Morton G., Cho A.K., Fairbanks L.A. (2008). Long-term methamphetamine administration in the vervet monkey models aspects of a human exposure: Brain neurotoxicity and behavioral profiles. Neuropsychopharmacology.

[40] Melega W.P., Raleigh M.J., Stout D.B., Lacan G., Huang S.C., Phelps M.E. (1997). Recovery of striatal dopamine function after acute amphetamine- and methamphetamine-induced neurotoxicity in the vervet monkey. Brain Res..

[41] Fumagalli F., Gainetdinov R.R., Valenzano K.J., Caron M.G. (1998). Role of dopamine transporter in methamphetamine-induced neurotoxicity: Evidence from mice lacking the transporter. J. Neurosci..

[42] Zhu J.P., Xu W., Angulo J.A. (2005). Disparity in the temporal appearance of methamphetamine-induced apoptosis and depletion of dopamine terminal markers in the striatum of mice. Brain Res..

[43] Anderson K.L., Itzhak Y. (2006). Methamphetamine-induced selective dopaminergic neurotoxicity is accompanied by an increase in striatal nitrate in the mouse. Ann. N. Y. Acad. Sci..

[44] Fornai F., Bassi L., Torracca M.T., Scalori V., Corsini G.U. (1995). Norepinephrine loss exacerbates methamphetamine-induced striatal dopamine depletion in mice. Eur. J. Pharmacol..

[45] Kita T., Paku S., Takahashi M., Kubo K., Wagner G.C., Nakashima T. (1998). Methamphetamine-induced neurotoxicity in BALB/c, DBA/2N and C57BL/6N mice. Neuropharmacology.

[46] Friedman S.D., Castaneda E., Hodge G.K. (1998). Long-term monoamine depletion, differential recovery, and subtle behavioral impairment following methamphetamine-induced neurotoxicity. Pharmacol. Biochem. Behav..

[47] Bakhit C., Morgan M.E., Peat M.A., Gibb J.W. (1981). Long-term effects of methamphetamine on the synthesis and metabolism of 5-hydroxytryptamine in various regions of the rat brain. Neuropharmacology.

[48] Graham D.L., Noailles P.A., Cadet J.L. (2008). Differential neurochemical consequences of an escalating dose-binge regimen followed by single-day multiple-dose methamphetamine challenges. J. Neurochem..

[49] Woolverton W.L., Ricaurte G.A., Forno L.S., Seiden L.S. (1989). Long-term effects of chronic methamphetamine administration in rhesus monkeys. Brain Res..

[50] Jayanthi S., Deng X., Ladenheim B., McCoy M.T., Cluster A., Cai N.S., Cadet J.L. (2005). Calcineurin/NFAT-induced up-regulation of the Fas ligand/Fas death pathway is involved in methamphetamine-induced neuronal apoptosis. Proc. Natl. Acad. Sci. USA.

[51] Kadota T., Kadota K. (2004). Neurotoxic morphological changes induced in the medial prefrontal cortex of rats behaviorally sensitized to methamphetamine. Arch. Histol. Cytol..

[52] Commins D.L., Seiden L.S. (1986). alpha-Methyltyrosine blocks methylamphetamine-induced degeneration in the rat somatosensory cortex. Brain Res..

[53] Eisch A.J., Marshall J.F. (1998). Methamphetamine neurotoxicity: Dissociation of striatal dopamine terminal damage from parietal cortical cell body injury. Synapse.

[54] O’Dell S.J., Marshall J.F. (2000). Repeated administration of methamphetamine damages cells in the somatosensory cortex: Overlap with cytochrome oxidase-rich barrels. Synapse.

[55] Kuczenski R., Everall I.P., Crews L., Adame A., Grant I., Masliah E. (2007). Escalating dose-multiple binge methamphetamine exposure results in degeneration of the neocortex and limbic system in the rat. Exp. Neurol..

[56] Deng X., Ladenheim B., Jayanthi S., Cadet J.L. (2007). Methamphetamine administration causes death of dopaminergic neurons in the mouse olfactory bulb. Biol. Psychiatry.

[57] Deng X., Ladenheim B., Tsao L.I., Cadet J.L. (1999). Null mutation of c-fos causes exacerbation of methamphetamine-induced neurotoxicity. J. Neurosci..

[58] Deng X., Wang Y., Chou J., Cadet J.L. (2001). Methamphetamine causes widespread apoptosis in the mouse brain: Evidence from using an improved TUNEL histochemical method. Brain Res. Mol. Brain Res..

[59] Schmued L.C., Bowyer J.F. (1997). Methamphetamine exposure can produce neuronal degeneration in mouse hippocampal remnants. Brain Res..

[60] Zhu J.P., Xu W., Angulo N., Angulo J.A. (2006). Methamphetamine-induced striatal apoptosis in the mouse brain: Comparison of a binge to an acute bolus drug administration. Neurotoxicology.

[61] Cadet J.L., Jayanthi S., Deng X. (2005). Methamphetamine-induced neuronal apoptosis involves the activation of multiple death pathways. Review. Neurotox Res..

[62] Cadet J.L., Krasnova I.N., Jayanthi S., Lyles J. (2007). Neurotoxicity of substituted amphetamines: Molecular and cellular mechanisms. Neurotox Res..

[63] Cadet J.L., Brannock C. (1998). Free radicals and the pathobiology of brain dopamine systems. Neurochem. Int..

[64] Graham D.G., Tiffany S.M., Bell W.R., Gutknecht W.F. (1978). Autoxidation *versus* covalent binding of quinones as the mechanism of toxicity of dopamine, 6-hydroxydopamine, and related compounds toward C1300 neuroblastoma cells *in vitro*. Mol. Pharmacol..

[65] Cohen G. (1987). Monoamine oxidase, hydrogen peroxide, and Parkinson’s disease. Adv. Neurol..

[66] Mello Filho A.C., Meneghini R. (1984). *In vivo* formation of single-strand breaks in DNA by hydrogen peroxide is mediated by the Haber-Weiss reaction. Biochim. Biophys. Acta.

[67] Potashkin J.A., Meredith G.E. (2006). The role of oxidative stress in the dysregulation of gene expression and protein metabolism in neurodegenerative disease. Antioxid. Redox Signal..

[68] Sonsalla P.K., Gibb J.W., Hanson G.R. (1986). Roles of D1 and D2 dopamine receptor subtypes in mediating the methamphetamine-induced changes in monoamine systems. J. Pharmacol. Exp. Ther..

[69] Albers D.S., Sonsalla P.K. (1995). Methamphetamine-induced hyperthermia and dopaminergic neurotoxicity in mice: Pharmacological profile of protective and nonprotective agents. J. Pharmacol. Exp. Ther..

[70] Kuhn D.M., Francescutti-Verbeem D.M., Thomas D.M. (2008). Dopamine disposition in the presynaptic process regulates the severity of methamphetamine-induced neurotoxicity. Ann. N. Y. Acad. Sci..

[71] Thomas D.M., Francescutti-Verbeem D.M., Kuhn D.M. (2008). The newly synthesized pool of dopamine determines the severity of methamphetamine-induced neurotoxicity. J. Neurochem..

[72] Wagner G.C., Lucot J.B., Schuster C.R., Seiden L.S. (1983). Alpha-methyltyrosine attenuates and reserpine increases methamphetamine-induced neuronal changes. Brain Res..

[73] Ricaurte G.A., Fuller R.W., Perry K.W., Seiden L.S., Schuster C.R. (1983). Fluoxetine increases long-lasting neostriatal dopamine depletion after administration of *d*-methamphetamine and *d*-amphetamine. Neuropharmacology.

[74] Schmidt C.J., Gibb J.W. (1985). Role of the serotonin uptake carrier in the neurochemical response to methamphetamine: Effects of citalopram and chlorimipramine. Neurochem. Res..

[75] Abekawa T., Ohmori T., Koyama T. (1994). Effects of repeated administration of a high dose of methamphetamine on dopamine and glutamate release in rat striatum and nucleus accumbens. Brain Res..

[76] Sonsalla P.K., Nicklas W.J., Heikkila R.E. (1989). Role for excitatory amino acids in methamphetamine-induced nigrostriatal dopaminergic toxicity. Science.

[77] Chung K.K., Dawson T.M., Dawson V.L. (2005). Nitric oxide, *S*-nitrosylation and neurodegeneration. Cell. Mol. Biol. (Noisy-le-Grand).

[78] Pacher P., Beckman J.S., Liaudet L. (2007). Nitric oxide and peroxynitrite in health and disease. Physiol. Rev..

[79] Imam S.Z., Newport G.D., Itzhak Y., Cadet J.L., Islam F., Slikker W., Ali S.F. (2001). Peroxynitrite plays a role in methamphetamine-induced dopaminergic neurotoxicity: Evidence from mice lacking neuronal nitric oxide synthase gene or overexpressing copper-zinc superoxide dismutase. J. Neurochem..

[80] Itzhak Y., Ali S.F. (2006). Role of nitrergic system in behavioral and neurotoxic effects of amphetamine analogs. Pharmacol. Ther..

[81] LaVoie M.J., Hastings T.G. (1999). Dopamine quinone formation and protein modification associated with the striatal neurotoxicity of methamphetamine: Evidence against a role for extracellular dopamine. J. Neurosci..

[82] Yuan J., Darvas M., Sotak B., Hatzidimitriou G., McCann U.D., Palmiter R.D., Ricaurte G.A. (2010). Dopamine is not essential for the development of methamphetamine-induced neurotoxicity. J. Neurochem..

[83] Krasnova I.N., Cadet J.L. (2009). Methamphetamine toxicity and messengers of death. Brain Res. Rev..

[84] Sharma H.S., Sjoquist P.O., Ali S.F. (2007). Drugs of abuse-induced hyperthermia, blood-brain barrier dysfunction and neurotoxicity: Neuroprotective effects of a new antioxidant compound H-290/51. Curr. Pharm. Des..

[85] Block M.L., Zecca L., Hong J.S. (2007). Microglia-mediated neurotoxicity: Uncovering the molecular mechanisms. Nat. Rev. Neurosci..

[86] Raivich G. (2005). Like cops on the beat: The active role of resting microglia. Trends Neurosci..

[87] Sekine Y., Ouchi Y., Sugihara G., Takei N., Yoshikawa E., Nakamura K., Iwata Y., Tsuchiya K.J., Suda S. (2008). Methamphetamine causes microglial activation in the brains of human abusers. J. Neurosci..

[88] LaVoie M.J., Card J.P., Hastings T.G. (2004). Microglial activation precedes dopamine terminal pathology in methamphetamine-induced neurotoxicity. Exp. Neurol..

[89] Moszczynska A., Fitzmaurice P., Ang L., Kalasinsky K.S., Schmunk G.A., Peretti F.J., Aiken S.S., Wickham D.J., Kish S.J. (2004). Why is parkinsonism not a feature of human methamphetamine users?. Brain.

[90] Wilson J.M., Kalasinsky K.S., Levey A.I., Bergeron C., Reiber G., Anthony R.M., Schmunk G.A., Shannak K., Haycock J.W., Kish S.J. (1996). Striatal dopamine nerve terminal markers in human, chronic methamphetamine users. Nat. Med..

[91] Schiffer W.K., Lee D.E., Brodie J.D., Dewey S.L. (2005). Imaging addiction with PET: Is insight in sight?. Drug Discov. Today.

[92] Hou H., Tian M., Zhang H. (2012). Positron emission tomography molecular imaging of dopaminergic system in drug addiction. Anat. Rec. (Hoboken).

[93] Gatley S.J., Volkow N.D. (1998). Addiction and imaging of the living human brain. Drug Alcohol Depend..

[94] Volkow N.D., Chang L., Wang G.J., Fowler J.S., Ding Y.S., Sedler M., Logan J., Franceschi D., Gatley J., Hitzemann R. (2001). Low level of brain dopamine D2 receptors in methamphetamine abusers: Association with metabolism in the orbitofrontal cortex. Am. J. Psychiatry.

[95] Volkow N.D., Chang L., Wang G.J., Fowler J.S., Franceschi D., Sedler M.J., Gatley S.J., Hitzemann R., Ding Y.S., Wong C., Logan J. (2001). Higher cortical and lower subcortical metabolism in detoxified methamphetamine abusers. Am. J. Psychiatry.

[96] Iyo M., Nishio M., Itoh T., Fukuda H., Suzuki K., Yamasaki T., Fukui S., Tateno Y. (1993). Dopamine D2 and serotonin S2 receptors in susceptibility to methamphetamine psychosis detected by positron emission tomography. Psychiatry Res..

[97] McCann U.D., Wong D.F., Yokoi F., Villemagne V., Dannals R.F., Ricaurte G.A. (1998). Reduced striatal dopamine transporter density in abstinent methamphetamine and methcathinone users: Evidence from positron emission tomography studies with [11C]WIN-35,428. J. Neurosci..

[98] Volkow N.D., Chang L., Wang G.J., Fowler J.S., Leonido-Yee M., Franceschi D., Sedler M.J., Gatley S.J., Hitzemann R., Ding Y.S. (2001). Association of dopamine transporter reduction with psychomotor impairment in methamphetamine abusers. Am. J. Psychiatry.

[99] Volkow N.D., Chang L., Wang G.J., Fowler J.S., Franceschi D., Sedler M., Gatley S.J., Miller E., Hitzemann R., Ding Y.S., Logan J. (2001). Loss of dopamine transporters in methamphetamine abusers recovers with protracted abstinence. J. Neurosci..

[100] Sekine Y., Iyo M., Ouchi Y., Matsunaga T., Tsukada H., Okada H., Yoshikawa E., Futatsubashi M., Takei N., Mori N. (2001). Methamphetamine-related psychiatric symptoms and reduced brain dopamine transporters studied with PET. Am. J. Psychiatry.

[101] Sekine Y., Minabe Y., Ouchi Y., Takei N., Iyo M., Nakamura K., Suzuki K., Tsukada H., Okada H., Yoshikawa E. (2003). Association of dopamine transporter loss in the orbitofrontal and dorsolateral prefrontal cortices with methamphetamine-related psychiatric symptoms. Am. J. Psychiatry.

[102] Wang G.-J., Volkow N.D., Chang L., Miller E., Sedler M., Hitzemann R., Zhu W., Logan J., Ma Y., Fowler J.S. (2004). Partial recovery of brain metabolism in methamphetamine abusers after protracted abstinence. Am. J. Psychiatry.

[103] London E.D., Simon S.L., Berman S.M., Mandelkern M.A., Lichtman A.M., Bramen J., Shinn A.K., Miotto K., Learn J., Dong Y. (2004). Mood disturbances and regional cerebral metabolic abnormalities in recently abstinent methamphetamine abusers. Arch. Gen. Psychiatry.

[104] London E.D., Berman S.M., Voytek B., Simon S.L., Mandelkern M.A., Monterosso J., Thompson P.M., Brody A.L., Geaga J.A., Hong M.S. (2005). Cerebral metabolic dysfunction and impaired vigilance in recently abstinent methamphetamine abusers. Biol. Psychiatry.

[105] Kim S.J., Lyoo I.K., Hwang J., Sung Y.H., Lee H.Y., Lee D.S., Jeong D.-U., Renshaw P.F. (2005). Frontal glucose hypometabolism in abstinent methamphetamine users. Neuropsychopharmacology.

[106] Johanson C.E., Frey K.A., Lundahl L.H., Keenan P., Lockhart N., Roll J., Galloway G.P., Koeppe R.A., Kilbourn M.R., Robbins T., Schuster C.R. (2006). Cognitive function and nigrostriatal markers in abstinent methamphetamine abusers. Psychopharmacology.

[107] Sekine Y., Ouchi Y., Takei N., Yoshikawa E., Nakamura K., Futatsubashi M., Okada H., Minabe Y., Suzuki K., Iwata Y. (2006). Brain serotonin transporter density and aggression in abstinent methamphetamine abusers. Arch. Gen. Psychiatry.

[108] Berman S.M., Voytek B., Mandelkern M.A., Hassid B.D., Isaacson A., Monterosso J., Miotto K., Ling W., London E.D. (2008). Changes in cerebral glucose metabolism during early abstinence from chronic methamphetamine abuse. Mol. Psychiatry.

[109] Boileau I., Rusjan P., Houle S., Wilkins D., Tong J., Selby P., Guttman M., Saint-Cyr J.A., Wilson A.A., Kish S.J. (2008). Increased vesicular monoamine transporter binding during early abstinence in human methamphetamine users: Is VMAT2 a stable dopamine neuron biomarker?. J. Neurosci..

[110] Lee B., London E.D., Poldrack R.A., Farahi J., Nacca A., Monterosso J.R., Mumford J.A., Bokarius A.V., Dahlbom M., Mukherjee J. (2009). Striatal dopamine D_2_/D_3_ receptor availability is reduced in methamphetamine dependence and is linked to impulsivity. J. Neurosci..

[111] Boileau I., Houle S., Rusjan P.M., Furukawa Y., Wilkins D., Tong J., Selby P., Wilson A.A., Kish S.J. (2010). Influence of a low dose of amphetamine on vesicular monoamine transporter binding: A PET (+)[11C]DTBZ study in humans. Synapse.

[112] Wang G.J., Smith L., Volkow N.D., Telang F., Logan J., Tomasi D., Wong C.T.,  Hoffman W., Jayne M., Alia-Klein N. (2011). Decreased dopamine activity predicts relapse in methamphetamine abusers. Mol. Psychiatry.

[113] Boileau I., Payer D., Houle S., Behzadi A., Rusjan P.M., Tong J., Wilkins D., Selby P., George T.P., Zack M. (2012). Higher binding of the dopamine D3 receptor-preferring ligand [11C]-(+)-propyl-hexahydro-naphtho-oxazin in methamphetamine polydrug users: A positron emission tomography study. J. Neurosci..

[114] Mawlawi O., Martinez D., Slifstein M., Broft A., Chatterjee R., Hwang D.R., Huang Y., Simpson N., Ngo K., van Heertum R., Laruelle M. (2001). Imaging human mesolimbic dopamine transmission with positron emission tomography: I. Accuracy and precision of D(2) receptor parameter measurements in ventral striatum. J. Cereb. Blood Flow Metab..

[115] Volkow N.D., Fowler J.S., Wang G.J., Hitzemann R., Logan J., Schlyer D. J., Dewey S.L., Wolf A.P. (1993). Decreased dopamine D2 receptor availability is associated with reduced frontal metabolism in cocaine abusers. Synapse.

[116] Volkow N.D., Wang G.J., Fowler J.S., Logan J., Gatley S.J., Hitzemann R., Chen A.D., Dewey S.L., Pappas N. (1997). Decreased striatal dopaminergic responsiveness in detoxified cocaine-dependent subjects. Nature.

[117] Ashby C.R., Wang R.Y. (1990). Effects of antipsychotic drugs on 5-HT2 receptors in the medial prefrontal cortex: Microiontophoretic studies. Brain Res..

[118] Kish S.J. (2008). Pharmacologic mechanisms of crystal meth. CMAJ.

[119] Murray A.M., Ryoo H.L., Gurevich E., Joyce J.N. (1994). Localization of dopamine D3 receptors to mesolimbic and D2 receptors to mesostriatal regions of human forebrain. Proc. Natl. Acad. Sci. USA.

[120] Sokoloff P., Giros B., Martres M., Bouthenet M.L., Schwartz J.C. (1990). Molecular cloning and characterization of a novel dopamine receptor (D3) as a target for neuroleptics. Nature.

[121] Mash D. (1997). CD3 receptor binding in human brain during cocaine overdose. Mol. Psychiatry.

[122] Segal D.M., Moraes C.T., Mash D.C. (1997). Up-regulation of D3 dopamine receptor mRNA in the nucleus accumbens of human cocaine fatalities. Brain Res. Mol. Brain Res..

[123] Sokoloff P., le Foll B., Perachon S., Bordet R., Ridray S., Schwartz J.C. (2001). The dopamine D3 receptor and drug addiction. Neurotox Res..

[124] Staley J.K., Mash D.C. (1996). Adaptive increase in D3 dopamine receptors in the brain reward circuits of human cocaine fatalities. J. Neurosci..

[125] Narendran R., Slifstein M., Guillin O., Hwang Y., Hwang D.R., Scher E., Reeder S., Rabiner E., Laruelle M. (2006). Dopamine (D2/3) receptor agonist positron emission tomography radiotracer [11C]-(+)-PHNO is a D3 receptor preferring agonist *in vivo*. Synapse.

[126] Ernst T., Chang L., Leonido-Yee M., Speck O. (2000). Evidence for long-term neurotoxicity associated with methamphetamine abuse: A 1H MRS study. Neurology.

[127] Scheel-Kruger J. (1986). Dopamine-GABA interactions: Evidence that GABA transmits, modulates and mediates dopaminergic functions in the basal ganglia and the limbic system. Acta Neurol. Scand. Suppl..

[128] Gibb J.W., Johnson M., Elayan I., Lim H.K., Matsuda L., Hanson G.R. (1997). Neurotoxicity of amphetamines and their metabolites. NIDA Res. Monogr..

[129] Seiden L.S., Sabol K.E. (1996). Methamphetamine and methylenedioxymethamphetamine neurotoxicity: Possible mechanisms of cell destruction. NIDA Res. Monogr..

[130] Schwartz W.J., Smith C.B., Davidsen L., Savaki H., Sokoloff L., Mata M., Fink D.J., Gainer H. (1979). Metabolic mapping of functional activity in the hypothalamo-neurohypophysial system of the rat. Science.

[131] Pu C., Broening H.W., Vorhees C.V. (1996). Effect of methamphetamine on glutamate-positive neurons in the adult and developing rat somatosensory cortex. Synapse.

[132] Kalechstein A.D., Newton T.F., Green M. (2003). Methamphetamine dependence is associated with neurocognitive impairment in the initial phases of abstinence. J. Neuropsychiatry Clin. Neurosci..

[133] Dluzen D.E., McDermott J.L. (2002). Estrogen, anti-estrogen, and gender: Differences in methamphetamine neurotoxicity. Ann. N. Y. Acad. Sci..

[134] Bonson K.R., Grant S.J., Contoreggi C.S., Links J.M., Metcalfe J., Weyl H.L., Kurian V., Ernst M., London E.D. (2002). Neural systems and cue-induced cocaine craving. Neuropsychopharmacology.

[135] Childress A.R., Mozley P.D., McElgin W., Fitzgerald J., Reivich M., O’Brien C.P. (1999). Limbic activation during cue-induced cocaine craving. Am. J. Psychiatry.

[136] Garavan H., Pankiewicz J., Bloom A., Cho J.K., Sperry L., Ross T.J., Salmeron B.J., Risinger R., Kelley D., Stein E.A. (2000). Cue-induced cocaine craving: Neuroanatomical specificity for drug users and drug stimuli. Am. J. Psychiatry.

[137] Kilts C.D., Schweitzer J.B., Quinn C.K., Gross R.E., Faber T.L., Muhammad F., Ely T.D., Hoffman J.M., Drexler K.P. (2001). Neural activity related to drug craving in cocaine addiction. Arch. Gen. Psychiatry.

[138] Maas L.C., Lukas S.E., Kaufman M.J., Weiss R.D., Daniels S.L., Rogers V.W., Kukes T.J., Renshaw P.F. (1998). Functional magnetic resonance imaging of human brain activation during cue-induced cocaine craving. Am. J. Psychiatry.

[139] Wang G.J., Volkow N.D., Fowler J.S., Cervany P., Hitzemann R.J., Pappas N.R., Wong C.T., Felder C. (1999). Regional brain metabolic activation during craving elicited by recall of previous drug experiences. Life Sci..

[140] Crino P.B., Morrison J.H., Hof P.R., Vogt B.A., Gabriel M. (1993). Monoaminergic Innervation of Cingulate Cortex. Neurobiology of Cingulate Cortex and Limbic Thalamus: A Comprehensive Handbook.

[141] Ogawa S., Tank D.W., Menon R., Ellermann J.M., Kim S.G., Merkle H., Ugurbil K. (1992). Intrinsic signal changes accompanying sensory stimulation: Functional brain mapping with magnetic resonance imaging. Proc. Natl. Acad. Sci. USA.

[142] Smith K. (2012). Brain imaging: fMRI 2.0. Nature.

[143] Ogawa S., Lee T.M., Kay A.R., Tank D.W. (1990). Brain magnetic resonance imaging with contrast dependent on blood oxygenation. Proc. Natl. Acad. Sci. USA.

[144] Salo R., Fassbender C. (2012). Structural, functional and spectroscopic MRI studies of methamphetamine addiction. Curr. Top. Behav. Neurosci..

[145] Attwell D., Iadecola C. (2002). The neural basis of functional brain imaging signals. Trends Neurosci..

[146] Fox P.T., Raichle M.E. (1986). Focal physiological uncoupling of cerebral blood flow and oxidative metabolism during somatosensory stimulation in human subjects. Proc. Natl. Acad. Sci. USA.

[147] Malonek D., Dirnagl U., Lindauer U., Yamada K., Kanno I., Grinvald A. (1997). Vascular imprints of neuronal activity: Relationships between the dynamics of cortical blood flow, oxygenation, and volume changes following sensory stimulation. Proc. Natl. Acad. Sci. USA.

[148] Paulus M.P., Hozack N., Frank L., Brown G., Schuckit M.A. (2003). Decision making by methamphetamine-dependent subjects is associated with error-rate-independent decrease in prefrontal and parietal activation. Biol. Psychiatry.

[149] Paulus M.P., Tapert S.F., Schuckit M.A. (2005). Neural activation patterns of methamphetamine-dependent subjects during decision making predict relapse. Arch. Gen. Psychiatry.

[150] Monterosso J.R., Ainslie G., Xu J., Cordova X., Domier C.P., London E.D. (2007). Frontoparietal cortical activity of methamphetamine-dependent and comparison subjects performing a delay discounting task. Hum. Brain Mapp..

[151] Hoffman W.F., Schwartz D.L., Huckans M.S., McFarland B.H., Meiri G., Stevens A.A., Mitchell S.H. (2008). Cortical activation during delay discounting in abstinent methamphetamine dependent individuals. Psychopharmacology.

[152] Leland D.S., Arce E., Miller D.A., Paulus M.P. (2008). Anterior cingulate cortex and benefit of predictive cueing on response inhibition in stimulant dependent individuals. Biol. Psychiatry.

[153] Salo R., Ursu S., Buonocore M.H., Leamon M.H., Carter C. (2009). Impaired prefrontal cortical function and disrupted adaptive cognitive control in methamphetamine abusers: A functional magnetic resonance imaging study. Biol. Psychiatry.

[154] Nestor L.J., Ghahremani D.G., Monterosso J., London E.D. (2011). Prefrontal hypoactivation during cognitive control in early abstinent methamphetamine-dependent subjects. Psychiatry Res..

[155] Paulus M. P., Hozack N., Zauscher B., McDowell J.E., Frank L., Brown G.G., Braff D.L. (2001). Prefrontal, parietal, and temporal cortex networks underlie decision-making in the presence of uncertainty. Neuroimage.

[156] Cardinal R.N. (2006). Neural systems implicated in delayed and probabilistic reinforcement. Neural. Netw..

[157] Steinglass J.E., Figner B., Berkowitz S., Simpson H.B., Weber E.U., Walsh B.T. (2012). Increased capacity to delay reward in anorexia nervosa. J. Int. Neuropsychol. Soc..

[158] Kirby K.N., Petry N.M. (2004). Heroin and cocaine abusers have higher discount rates for delayed rewards than alcoholics or non-drug-using controls. Addiction.

[159] MacKillop J., Amlung M.T., Few L.R., Ray L.A., Sweet L., Munafo M.R. (2011). Delayed reward discounting and addictive behavior: A meta-analysis. Psychopharmacology.

[160] Bickel W.K., Madden G.J., Petry N.M. (1998). The price of change: The behavioral economics of drug dependence. Behav. Ther..

[161] Ersche K.D., Fletcher P.C., Lewis S.J., Clark L., Stocks-Gee G., London M., Deakin J.B., Robbins T.W., Sahakian B.J. (2005). Abnormal frontal activations related to decision-making in current and former amphetamine and opiate dependent individuals. Psychopharmacology.

[162] Kaufman J.N., Ross T.J., Stein E.A., Garavan H. (2003). Cingulate hypoactivity in cocaine users during a GO-NOGO task as revealed by event-related functional magnetic resonance imaging. J. Neurosci..

[163] Kerns J.G., Cohen J.D., MacDonald A.W., Cho R.Y., Stenger V.A., Carter C.S. (2004). Anterior cingulate conflict monitoring and adjustments in control. Science.

[164] MacDonald A.W., Cohen J.D., Stenger V.A., Carter C.S. (2000). Dissociating the role of the dorsolateral prefrontal and anterior cingulate cortex in cognitive control. Science.

[165] Carp J., Kim K., Taylor S.F., Fitzgerald K.D., Weissman D.H. (2010). Conditional differences in mean reaction time explain effects of response congruency, but not accuracy, on posterior medial frontal cortex activity. Front. Hum. Neurosci..

[166] Menon V., Adleman N.E., White C.D., Glover G.H., Reiss A.L. (2001). Error-related brain activation during a Go/NoGo response inhibition task. Hum. Brain Map..

[167] Botvinick M., Nystrom L.E., Fissell K., Carter C.S., Cohen J.D. (1999). Conflict monitoring *versus* selection-for-action in anterior cingulate cortex. Nature.

[168] Ridderinkhof K.R., Ullsperger M., Crone E.A., Nieuwenhuis S. (2004). The role of the medial frontal cortex in cognitive control. Science.

[169] Ridderinkhof K.R., van den Wildenberg W.P., Segalowitz S.J., Carter C.S. (2004). Neurocognitive mechanisms of cognitive control: The role of prefrontal cortex in action selection, response inhibition, performance monitoring, and reward-based learning. Brain Cogn..

[170] Aron A.R., Fletcher P.C., Bullmore E.T., Sahakian B.J., Robbins T.W. (2003). Stop-signal inhibition disrupted by damage to right inferior frontal gyrus in humans. Nat. Neurosci..

[171] Bunge S.A., Dudukovic N.M., Thomason M.E., Vaidya C.J., Gabrieli J.D. (2002). Immature frontal lobe contributions to cognitive control in children: Evidence from fMRI. Neuron.

[172] Hoshi E., Tanji J. (2004). Differential roles of neuronal activity in the supplementary and presupplementary motor areas: From information retrieval to motor planning and execution. J. Neurophysiol..

[173] Hester R., Fassbender C., Garavan H. (2004). Individual differences in error processing: A review and reanalysis of three event-related fMRI studies using the GO/NOGO task. Cereb. Cortex.

[174] Wunderlich K., Rangel A., O’Doherty J.P. (2009). Neural computations underlying action-based decision making in the human brain. Proc. Natl. Acad. Sci. USA.

[175] Botvinick M.M., Braver T.S., Barch D.M., Carter C.S., Cohen J.D. (2001). Conflict monitoring and cognitive control. Psychol. Rev..

[176] Botvinick M.M., Cohen J.D., Carter C.S. (2004). Conflict monitoring and anterior cingulate cortex: An update. Trends Cogn. Sci..

[177] Carter C.S., Braver T.S., Barch D.M., Botvinick M.M., Noll D., Cohen J.D. (1998). Anterior cingulate cortex, error detection, and the online monitoring of performance. Science.

[178] Garavan H., Ross T.J., Murphy K., Roche R.A., Stein E.A. (2002). Dissociable executive functions in the dynamic control of behavior: Inhibition, error detection, and correction. Neuroimage.

[179] Magno E., Foxe J.J., Molholm S., Robertson I.H., Garavan H. (2006). The anterior cingulate and error avoidance. J. Neurosci..

[180] Naqvi N.H., Bechara A. (2009). The hidden island of addiction: The insula. Trends Neurosci..

[181] Birn R.M., Cox R.W., Bandettini P.A. (2002). Detection *versus* estimation in event-related fMRI: Choosing the optimal stimulus timing. Neuroimage.

[182] Egner T. (2008). Multiple conflict-driven control mechanisms in the human brain. Trends Cogn. Sci..

[183] Simon S.L., Dean A.C., Cordova X., Monterosso J.R., London E.D. (2010). Methamphetamine dependence and neuropsychological functioning: Evaluating change during early abstinence. J. Stud. Alcohol Drugs.

[184] Chang L., Cloak C., Patterson K., Grob C., Miller E.N., Ernst T. (2005). Enlarged striatum in abstinent methamphetamine abusers: A possible compensatory response. Biol. Psychiatry.

[185] Schlaepfer T.E., Lancaster E., Heidbreder R., Strain E.C., Kosel M., Fisch H.U., Pearlson G.D. (2006). Decreased frontal white-matter volume in chronic substance abuse. Int. J. Neuropsychopharmacol..

[186] Thompson P.M., Hayashi K.M., Simon S.L., Geaga J.A., Hong M.S., Sui Y., Lee J.Y., Toga A.W., Ling W., London E.D. (2004). Structural abnormalities in the brains of human subjects who use methamphetamine. J. Neurosci..

[187] Schwartz D.L., Mitchell A.D., Lahna D.L., Luber H.S., Huckans M.S., Mitchell S.H., Hoffman W.F. (2010). Global and local morphometric differences in recently abstinent methamphetamine-dependent individuals. Neuroimage.

[188] Oh J.S., Lyoo I.K., Sung Y.H., Hwang J., Kim J., Chung A., Park K.S., Kim S.J., Renshaw P.F., Song I.C. (2005). Shape changes of the corpus callosum in abstinent methamphetamine users. Neurosci. Lett..

[189] Jernigan T.L., Gamst A.C., Archibald S.L., Fennema-Notestine C., Mindt M.R., Marcotte T.D., Heaton R.K., Ellis R.J., Grant I. (2005). Effects of methamphetamine dependence and HIV infection on cerebral morphology. Am. J. Psychiatry.

[190] Bae S.C., Lyoo I.K., Sung Y.H., Yoo J., Chung A., Yoon S.-J., Kim D.-J., Hwang J., Kim S.J., Renshaw P.F. (2006). Increased white matter hyperintensities in male methamphetamine abusers. Drug Alcohol Depend..

[191] Kim S.J., Lyoo I.K., Hwang J., Chung A., Hoon Sung Y., Kim J., Kwon D.-H., Chang K.H., Renshaw P.F. (2006). Prefrontal grey-matter changes in short-term and long-term abstinent methamphetamine abusers. Int. J. Neuropsychopharmacol..

[192] Chung A., Lyoo I.K., Kim S.J., Hwang J., Bae S.C., Sung Y.H., Sim M.E., Song I.C., Kim J., Chang K.H., Renshaw P.F. (2007). Decreased frontal white-matter integrity in abstinent methamphetamine abusers. Int. J. Neuropsychopharmacol..

[193] Nakama H., Chang L., Fein G., Shimotsu R., Jiang C.S., Ernst T. (2011). Methamphetamine users show greater than normal age-related cortical gray matter loss. Addiction.

[194] Thomas D.M., Francescutti-Verbeem D.M., Liu X., Kuhn D.M. (2004). Identification of differentially regulated transcripts in mouse striatum following methamphetamine treatment—An oligonucleotide microarray approach. J. Neurochem..

[195] Brody A.L., Mandelkern M.A., Jarvik M.E., Lee G.S., Smith E.C., Huang J.C., Bota R.G., Bartzokis G., London E.D. (2004). Differences between smokers and nonsmokers in regional gray matter volumes and densities. Biol. Psychiatry.

[196] Wrase J., Makris N., Braus D.F., Mann K., Smolka M.N., Kennedy D.N., Caviness V.S., Hodge S.M., Tang L., Albaugh M. (2008). Amygdala volume associated with alcohol abuse relapse and craving. Am. J. Psychiatry.

[197] Cole M.W., Schneider W. (2007). The cognitive control network: Integrated cortical regions with dissociable functions. Neuroimage.

[198] Hare T.A., Camerer C.F., Rangel A. (2009). Self-control in decision-making involves modulation of the vmPFC valuation system. Science.

[199] Fein G., Di Sclafani V. (2004). Cerebral reserve capacity: Implications for alcohol and drug abuse. Alcohol.

[200] Berman S., O’Neill J., Fears S., Bartzokis G., London E.D. (2008). Abuse of amphetamines and structural abnormalities in the brain. Ann. N. Y. Acad. Sci..

[201] Chang L., Ernst T., Speck O., Patel H., DeSilva M., Leonido-Yee M., Miller E.N. (2002). Perfusion MRI and computerized cognitive test abnormalities in abstinent methamphetamine users. Psychiatry Res..

[202] Paganini-Hill A., Ross R.K., Henderson B.E. (1988). Postmenopausal oestrogen treatment and stroke: A prospective study. BMJ.

[203] Schmidt R., Fazekas F., Reinhart B., Kapeller P., Fazekas G., Offenbacher H., Eber B., Schumacher M., Freidl W. (1996). Estrogen replacement therapy in older women: A neuropsychological and brain MRI study. J. Am. Geriatr. Soc..

[204] Myers R.E., Anderson L.I., Dluzen D.E. (2003). Estrogen, but not testosterone, attenuates methamphetamine-evoked dopamine output from superfused striatal tissue of female and male mice. Neuropharmacology.

